# Surface Modification of Gold Nanorods (GNRDs) Using Double Thermo-Responsive Block Copolymers: Evaluation of Self-Assembly and Stability of Nanohybrids

**DOI:** 10.3390/polym16233293

**Published:** 2024-11-26

**Authors:** Jesús E. Márquez-Castro, Angel Licea-Claverie, Carlos Guerrero-Sánchez, Eugenio R. Méndez

**Affiliations:** 1 Centro de Graduados e Investigación en Química, Tecnológico Nacional de México/Instituto Tecnológico de Tijuana, Tijuana 22454, Mexico; eduardo.mc@tectijuana.edu.mx; 2 Laboratory of Organic and Macromolecular Chemistry (IOMC), Friedrich Schiller University Jena, 07743 Jena, Germany; 3 Jena Center for Soft Matter (JCSM), Friedrich Schiller University Jena, 07743 Jena, Germany; 4 División de Física Aplicada, Centro de Investigación Científica y Educación Superior de Ensenada, Ensenada 22860, Mexico; emendez@cicese.edu.mx

**Keywords:** gold nanorods, RAFT polymerization, thermo-responsive polymers, photo-thermal therapy, nanohybrids

## Abstract

A series of copolymers containing a thermo-responsive biocompatible first block of poly[di(ethylene glycol) methyl ether methacrylate)-*co*-(oligo(ethylene glycol) methyl ether methacrylate], P(DEGMA-*co*-OEGMA) were chain-extended to incorporate either poly(*N*-isopropylacrylamide), PNIPAAm or poly(*N*-isopropylacrylamide-*co*-butyl acrylate), P(NIPAAm-co-BA) as second thermo-responsive block using reversible addition–fragmentation chain transfer (RAFT) polymerization. P(DEGMA-*co*-OEGMA)-*b*-PNIPAAm copolymers showed two response temperatures at 33 and 43 °C in an aqueous solution forming stable aggregates at 37 °C. In contrast, P(DEGMA-*co*-OEGMA)-*b*-P(NIPAAm-*co*-BA) copolymers showed aggregation below room temperature due to the shift in response temperature provoked by the presence of hydrophobic butyl acrylate (BA) units, and shrinkage upon heating up to body temperature, while maintaining the second response temperature above 40 °C. The terminal trithiocarbonate group of the block copolymers was modified to a thiol functionality and used to stabilize gold nanorods (GNRDs) via the “grafting to” approach. The Localized Surface Plasmon Resonance (LSPR) absorption band of GNRDs with an aspect ratio of 3.9 (length/diameter) was located at 820 nm after surface grafting with block copolymers showing a hydrodynamic diameter of 160 nm at 37 °C. On the other hand, the stability of the P(DEGMA-*co*-OEGMA)-*b*-PNIPAAm@GNRDs and P(DEGMA-*co*-OEGMA)-*b*-P(NIPAAm-*co*-BA)@GNRDs nanohybrids was monitored for 8 days; where the LSPR absorption band did not shift or show any broadening. Aqueous dispersed nanohybrids were irradiated with a near-infrared laser (300 mW), where the temperature of the surroundings increased 16 °C after 16 min, where conditions for no precipitation were determined. These tailored temperature-responsive nanohybrids represent interesting candidates to develop drug nanocarriers for photo-thermal therapies.

## 1. Introduction

Gold nanoparticles (AuNPs) display optical and electronic properties complementary to various applications due to their many advantages, such as compatibility, enhanced long thermo-stability, simple method of preparation, and multifunctionality [1,2], as well as their wavelength selective optical absorption. The development of AuNPs with different morphologies has received much attention in recent years, especially with the advent of plasmonic photo-thermal therapy (PPTT), also known as photo-thermal ablation, which employs these AuNPs as photo-thermic agents [3,4].

PPTT is currently one of the most promising research approaches in treating cancer cells and infectious diseases. The selective absorption properties of the photo-thermal agent are due to the excitation of localized surface plasmon resonance (LSPR), whose frequency is highly influenced by the size of the nanoparticles, their morphology, and the surrounding environment [5]. Among the diverse morphologies of AuNPs, special interest has been focused on gold nanorods (GNRDs) due to their outstanding physicochemical properties, such as light scattering, possibility to modulate their surface plasmon resonance and to couple to biomolecules or polymers via Au-S bonds, and their high surface-to-volume-ratio [6,7]. GNRDs exhibit the unique optical properties of strong light absorption and heat emission in the near-infrared (NIR) region (750–1000 nm) depending on their size and aspect ratio. However, GNRDs are cytotoxic due to the presence of hexadecyltrimethyl ammonium (CTAB) moieties on their surface, which are used to maintain dispersion and prevent aggregation [8,9].

To circumvent their cytotoxicity, researchers have used polymers, bioactive molecules, and phospholipid molecules to graft them onto the surface of GNRDs. In addition to the photo-therapeutic applications, NIR light-induced heating of GNRDs could also be utilized for drug and gene delivery applications, which are usually immobilized on the surface of GNRDs and loaded into the external concavities of functional polymer coatings [10,11]. On the other hand, due to the rather simple surface functionalization of GNRDs, a thermo-responsive copolymer coating can be introduced via a “grafting to” approach; commonly, GNRDs are grafted with polymers containing thiol (-SH)-reactive functional groups [12]. Polymers synthesized via reversible addition–fragmentation chain transfer (RAFT) polymerization are distinguished by the presence of functional end groups, such as trithiocarbonate, dithioester, or xanthate groups, which are derived from the chain transfer agent (CTA) used for their synthesis and can be transformed to thiol groups [13,14]. In turn, thiol-functionalized polymers can be conveniently used to decorate the surface of AuNPs via Au-S conjugation to form polymer@GNRDs nanohybrids [15], while some reports directly use trithiocarbonate terminated polymers for the same purpose [16].

Recently, thermo-responsive double hydrophilic block copolymers (TDHBCs) have gained a great deal of attention as a subclass of stimuli-responsive materials due to promising applications in the biomedical field, including drug delivery and stabilization of gold nanoparticles [17]. TDHBCs are composed of two blocks with different lower critical solution temperatures (LCST), such as poly(*N*-isopropylacrylamide)-*block*-poly(oligo ethylene glycol methyl ether acrylate) (PNIPAAm-*b*-POEGA) [18] and poly(diethylene glycol) methyl ether methacrylate)-*block*-poly(poly(ethylene glycol) methyl ether methacrylate) (PO_2_-*b*-PO_300_) [19]. In this context, PNIPAAm is probably the most extensively investigated thermo-responsive polymer in biomedical applications, whose LCST value in aqueous media is ca. 32 °C [20,21]. For example, for aqueous dispersions of PNIPAAm-*b*-POEGA, as the temperature rises above the LCST value of the PNIPAAm segment, the copolymer self-assembles to form micelles consisting of an insoluble core of PNIPAAm surrounded by a corona of a solvated POEGA block. As the temperature of the system further increases above the LCST of the POEGA segment, it becomes insoluble and collapses onto the surface of the PNIPAAm core [18]. The formation and dissociation of thermo-responsive diblock copolymers nano-assemblies can be controlled by temperature and, thus, without the need to use organic solvents [22,23]. Meanwhile, copolymers containing short oligo(ethylene glycol) methacrylates (OEGMAS) have also been identified for their multi-functionality as they exhibit both stimuli-responsive and biocompatible properties [24]. For instance, investigations on the poly[(di(ethylene glycol) methyl ether methacrylate)-*co*-(oligo(ethylene glycol)] methyl ether methacrylate)] (P(DEGMA-*co*-OEGMA)) copolymer system showed that the hydrophilic/hydrophobic thermal transition could be adjusted by varying the comonomer composition [25].

**Scheme 1:**
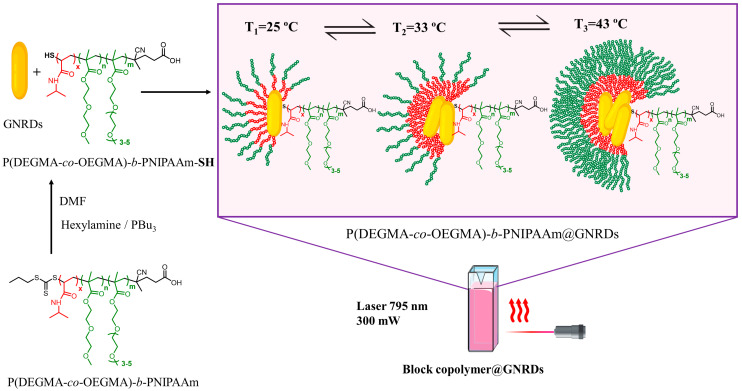
Schematic illustration of the preparation of P(DEGMA-*co*-OEGMA)-*b*-PNIPAAm@GNRDs nanohybrids.

Herein, we report the use of copolymers poly(DEGMA-*co*-OEGMA) with an adjusted cloud point temperature (*T_cp_*) prepared via RAFT polymerization using a trithiocarbonate RAFT agent (CTA) and chain-extended with NIPAAm and with NIPAAm/butyl acrylate (BA) comonomer mixtures to yield doubly thermo-responsive block copolymers. In addition, the self-assembly of these materials in aqueous media was evaluated against temperature. The *T_cp_* of the first block was designed to be above 40 °C by varying the comonomer content of P(DEGMA-*co*-OEGMA); similarly, the second *T_cp_* was adjusted to values below 33 °C using NIPAAm and BA as comonomers. Furthermore, the trithiocarbonate end-group in these copolymers was reduced using hexylamine, and the obtained thiol-terminated block copolymers were immobilized on the surface of GNRDs following a “grafting to” approach to yield block copolymer@GNRDs nanohybrids with a double thermo-responsive coating (see Scheme 1). Moreover, NIR-induced heating experiments on the nanohybrids in aqueous dispersions were carried out with the aid of a NIR laser diode (785 nm wavelength, output power up to 300 mW) where the behavior of self-assemblies as a function of temperature was also evaluated under physiological temperature conditions. The tailored temperature-dependent nanohybrids proposed herein may represent interesting candidates to develop drug loading and delivery strategies for medical therapies.

## 2. Materials and Methods

### 2.1. Materials

Oligo(ethylene glycol) methyl ether methacrylate (OEGMA, 99%, M_w_ = 300 g/mol), di(ethylene glycol) methyl ether methacrylate (DEGMA, 99%, M_w_ = 188.22 g/mol), and butyl acrylate (BA, 99%) were purified through a column packed with inhibitor removers for hydroquinone, while *N*-isopropylacrylamide (NIPAAm, 99%) was recrystallized in petroleum ether. 4,4-Azobis(4-cyano-valeric acid) (ACVA, 98%), tetrachloroauric acid trihydrate (HAuCl_4_.3H_2_O, 99%), sodium borohydride (NaBH_4_, 99%), ascorbic acid (C_6_H_8_O_6_, 99%), hexadecyltrimethylammonium bromide (CTAB, 99%), silver nitrate (AgNO_3_, 99%), sulfuric acid (H_2_SO_4_, 98%), n-hexylamine (C_6_H_15_N, 96%) and tributylphosphine [CH_3_(CH_2_)3P, 97%)] were used as received. Unless specified otherwise, all the chemicals used were acquired from Sigma-Aldrich (Toluca, Mexico). Petroleum ether, diethyl ether, dichloromethane (DCM), and dimethyl formamide (DMF) were purchased from FERMONT (Monterrey, Mexico). Purification of synthetic products was carried out via chromatography using silica gel (70–230 mesh, Across Organics, Geel, Belgium).

### 2.2. Synthesis of P(DEGMA-co-OEGMA) Copolymers

A series of copolymers (first block) were prepared using different ratios of monomer: CTA with a feed of DEGMA:OEGMA 70:30 mol% using 4-cyano-4-(propylthiocarbothioylthio) pentanoic acid as a trithiocarbonate-based CTA, which in turn was synthesized as described elsewhere [25]. The synthesis of the first block is briefly described: DEGMA (1.97 g, 10.5 mmol), OEGMA (1.35 g, 4.5 mmol), CTA (31 mg, 0.119 mmol), and ACVA (3.13 mg, 0.0199 mmol) were dissolved in dry DMF at 25% *v*/*v*, the mixture was placed into 20 mL glass ampoules containing a magnetic stir bar, argon was sparged into the solution for 25 min to displace oxygen and each ampoule was sealed. All reactions were carried out under magnetic stirring at 70 °C for 4 h. The polymerization was stopped by cooling to room temperature. Afterward, the copolymer was purified by precipitation into an excess of diethyl ether/petroleum ether (1:1 *v*/*v*) under constant agitation. Then, the copolymer was re-dissolved in the minimum amount of DCM and precipitated into an excess of diethyl ether (X3); the precipitate was recovered by discarding the supernatant, followed by drying under reduced pressure in a vacuum oven at room temperature.

### 2.3. Synthesis of P(DEGMA-co-OEGMA)-b-PNIPAAm Block Copolymers

The P(DEGMA-*co*-OEGMA) first blocks were used as macro-RAFT agents to incorporate the second thermo-responsive PNIPAAm blocks. The synthesis of one block copolymer is described in detail: P(DEGMA-*co*-OEGMA) (*M*_n GPC_ = 17,500 g mol^−1^) (0.200 g, 0.016 mmol), NIPAAm (115 mg, 1.025 mmol), and ACVA (0.286 mg, 0.00102 mmol) were dissolved in 2 mL of dry DMF. The mixture was placed into 10 mL glass ampoules containing a magnetic stir bar, argon was sparged into the solution for 25 min to displace oxygen and each ampoule was sealed; all reactions were carried out under magnetic stirring at 70 °C for 2 h. The polymerization was stopped by cooling to room temperature. Afterward, the diblock copolymer was purified by precipitation into an excess of diethyl ether/petroleum ether (1:1 *v*/*v*) under constant agitation. Then, the copolymer was re-dissolved in the minimum amount of DCM and precipitated into an excess of diethyl ether (X3), the precipitate was recovered by discarding the supernatant followed by drying under reduced pressure in a vacuum oven at room temperature.

### 2.4. Synthesis of P(DEGMA-co-OEGMA)-b-P(NIPAAm-co-BA)

A similar procedure was followed for the case of P(DEGMA-*co*-OEGMA)-*b*-P(NIPAAm-*co*-BA) block copolymers, i.e., utilizing P(DEGMA-*co*-OEGMA) as a macro-RAFT agent, with the difference that BA was added in amounts of 10 mol% with respect to NIPAAm. An example is described: P(DEGMA-*co*-OEGMA) (*M*_n SEC_ = 17,500 g mol^−1^) (0.200 g, 0.016 mmol), NIPAAm (102 mg, 0.923 mmol), BA (13 mg, 0.102 mmol), ACVA (0.286 mg, 0.00102 mmol) were dissolved in 2 mL of dry DMF. The mixture was placed into 10 mL glass ampoules containing a magnetic stir bar, argon was sparged into the solution for 25 min to displace oxygen and each ampoule was sealed. All reactions were carried out under magnetic stirring at 70 °C for 2 h. The polymerization was stopped by cooling to room temperature. Afterward, the diblock copolymer was purified by precipitation into an excess of diethyl ether/petroleum ether (1:1 *v*/*v*) under constant agitation. Then, the copolymer was re-dissolved in the minimum amount of DCM and precipitated into an excess of diethyl ether (X3), the precipitate was recovered by discarding the supernatant followed by drying under reduced pressure in a vacuum oven at room temperature.

### 2.5. Transformation of Trithiocarbonate End-Groups of the Block Copolymers into Thiol Functionality

The trithiocarbonate end-groups of the obtained copolymers were modified to thiols via aminolysis utilizing hexylamine adapting a reported methodology [26]. One example is described in detail: In a 20 mL Schlenk flask, (0.200 g, 0.0081 mmol) of P(DEGMA-*co*-OEGMA)-*b*-PNIPAAm (*M*_n SEC_ = 24,500 g mol^−1^) was dissolved in 2 mL of dry DMF, and the mixture was stirred for 20 min until homogenization, nitrogen was sparged into the solution for 25 min to displace oxygen. Then, hexylamine and tributyl phosphine were added into the reaction mixture in a molar ratio [hexylamine]:[copolymer-CTA]:[tributyl phosphine] of [50]:[1]:[10]. The mixture was stirred for 4 h and the color of the solution changed from pale yellow to transparent. Then, the copolymer-SH was purified by precipitation into an excess of diethyl ether/petroleum ether (1:1 *v*/*v*) under constant agitation. Then, the copolymer was re-dissolved in the minimum amount of DCM and precipitated into an excess of diethyl ether (X3), the precipitate was recovered by discarding the supernatant followed by drying under reduced pressure in a vacuum oven at room temperature. The product was obtained with a yield of approximately 85 wt%.

### 2.6. Synthesis and Stabilization of Gold Nanorods (GNRDs)

The GNRDs synthesis was performed using the seed-mediated growth method described elsewhere [26,27]. First, CTAB-coated gold seeds were obtained by chemical reduction of HAuCl_4_ utilizing NaBH_4_. Briefly, (0.25 mL, 10 mM) of HAuCl_4_ was slowly added into a CTAB solution (7.5 mL, 100 mM) under stirring. Then, (0.6 mL, 10 mM) NaBH_4_ was incorporated and the solution quickly changed from yellow to brown indicating the formation of gold seeds. Second, the GNRDs were prepared from gold seeds within a growth solution. The growth solution was prepared by adding (5 mL, 10 mM) of HAuCl_4,_ (100 mL, 10 mM) of CTAB, (1 mL, 10 mM) of AgNO_3_, and (1 mL, 1 M) of H_2_SO_4_ into a 250 mL round-bottom flask at 30 °C under stirring. Next, (0.8 mL, 100 mM) of ascorbic acid was added under stirring until the dispersion became colorless. Then, 0.250 mL of gold seed was added under vigorous stirring and left undisturbed for 6 h. The GNRDs were purified via centrifugation (10,000 rpm for 30 min) discarding the supernatant; this purification step was repeated twice. The purified GNRDs were dispersed in distilled water and stored in a refrigerated amber vial until use.

To graft P(DEGMA-*co*-OEGMA)-*b*-PNIPAAm-SH onto GNRDs, 5 mL of GNRDs in dispersion and 5 mg of copolymers were mixed under magnetic stirring for 3 h; the resulting dispersion was let to settle for 24 h, and precipitated GNRDs were removed by decantation as the product can be found in the supernatant. A similar procedure was followed in the case of P(DEGMA-*co*-OEGMA)-*b*-P(NIPAAm-*co*-BA)-SH; 5 mg of the copolymer was dissolved in DMF and slowly added into 5 mL of GNRDs dispersion. This mixture was dialyzed for 24 h, the resulting dispersion was let to settle for 24 h, and precipitated GNRDs were removed by decantation as the product can be found in the supernatant.

### 2.7. Characterization of Polymers

Proton nuclear magnetic resonance (^1^H-NMR) spectra were collected at 400 MHz on a Bruker AMX-400 (Bruker Corporation, Billerica, MA, USA) spectrometer at 298 K. Deuterated chloroform (CDCl_3_) was used as an analysis solvent for all samples. Chemical shifts are reported in parts per million (ppm) using tetramethylsilane (TMS) as the internal standard. Ultraviolet-visible (UV-Vis) spectra were recorded using a UV-Vis Varian Cary 100 spectrophotometer (Agilent Technologies, Santa Clara, CA, USA) in the wavelength range from 200 to 900 nm.

Size exclusion chromatography (SEC) was performed on a Waters 515 HPLC chromatograph (Waters Corp., Milford, MA, USA) equipped with a series of columns (Shodex: GF-1G, 7B, GF-510 HQ, GF-310 HQ, Tokyo, Japan), a Wyatt Technology refractive index detector (Optilab DSP, Santa Barbara, CA, USA) and a Wyatt Technology multiangle light scattering (LS) detector (Dawn DSP, Santa Barbara, CA, USA) equipped with a red laser (λ = 633 nm). Poly(ethylene oxide) standards of narrow dispersity (*Ð*) were used to check the accuracy of the LS detector in DMF as a mobile phase. DMF was used as the mobile phase at a 0.3 mL min^−1^ flow rate at 40 °C. Sample solutions were prepared using 20 mg mL^−1^ concentration and filtered through a 0.2 µm Nylon membrane.

### 2.8. Characterization of Aggregates and Nanohybrids

Dynamic light scattering (DLS) measurements were carried out using Malvern Instruments Nano-ZS Nanosizer (ZEN 3690), (Malvern, Worcestershire, UK). The instrument is equipped with a helium-neon laser (λ = 633 nm) with a size detection range of 0.6 nm to 5 μm. DLS experiments were performed at the scattering angle of 90°, and the distribution of sizes was calculated using Malvern instruments dispersion technology Zetasizer software (version 7.1) based on the CONTIN analysis and the Stokes–Einstein equation for spheres. The *D*_h_ reported is the average of three measurements.

The *T_cp_* was estimated via DLS using 1 mg mL^−1^ copolymer solutions and a temperature program that increased in two-degree steps with an equilibration time of 1 min between each step.

Morphological studies of GNRDs and nanohybrids block copolymer@GNRDs were performed using a H7500 transmission electron microscope (TEM) (Hitachi Co. Ltd., Tokyo, Japan), operating at an accelerating voltage of 80 kV. A drop of dispersed GNRDs or nanohybrids at a concentration value of 0.1 mg mL^−1^ was poured over a 75-mesh copper grid coated with a thin layer of carbon, followed by the removal of the excess liquid at room temperature.

Thermogravimetric analysis (TGA) was performed using TA-instruments Discovery-TGA equipment (TA-instrument, New Castle, DE, USA). Measurements were performed for block copolymers and nanohybrids block copolymer@GNRDs under a nitrogen atmosphere and by heating from room temperature up to 600 °C using a heating rate of 10 °C min^−1^.

### 2.9. NIR-Induced Heating Investigations on Nanohybrid Dispersions

Light-induced heating studies of nanohybrid dispersions were performed using a fiber-coupled diode laser (CNI, MDL-III-785, Changchun, China) with variable output power (a maximum of 300 mW) and wavelength of 785 nm. Two mL of nanohybrid dispersion was placed inside a quartz cell at room temperature without any kind of thermal isolation. A thermocouple was introduced into the sample to record temperature changes in intervals of 1 s for a total of 16 min.

## 3. Results and Discussion

### 3.1. Synthesis and Characterization of P(DEGMA-co-OEGMA) Macro-RAFT Agent

The synthesis of the P(DEGMA-*co*-OEGMA)-*b*-PNIPAAm and P(DEGMA-*co*-OEGMA)-*b*-P(NIPAAm-*co*-BA) block copolymers was accomplished by a two steps sequential RAFT polymerization procedure. A trithiocarbonate type CTA was chosen as it is known to produce well-defined polymers when used for the polymerization of methacrylates and acrylamides [28,29].

**Scheme 2:**
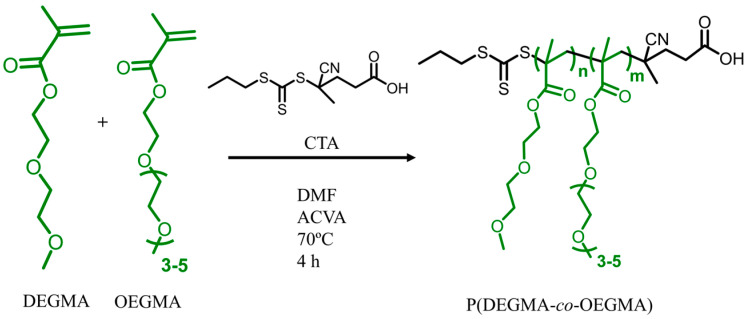
Synthesis route for P(DEGMA-*co*-OEGMA) copolymers vía RAFT polymerization.

**Figure 1:**
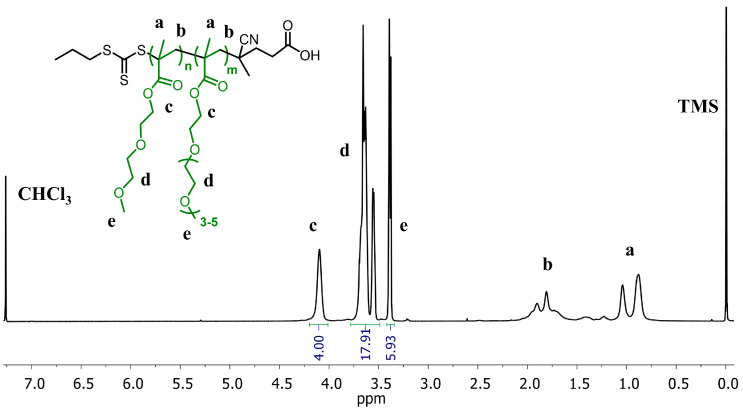
^1^H-NMR spectrum (400 MHz) in CDCl_3_ of the P(DEGMA-*co*-OEGMA) copolymer P2.

A series of P(DEGMA-*co*-OEGMA) copolymers of different molar masses were prepared keeping the DEGMA:OEGMA molar ratio constant at 70:30 in the feed (see Scheme 2). This particular comonomer composition was chosen to attain a LCST close to 40 °C [30]. The chemical structure and actual composition of the synthesized copolymers were determined by ^1^H-NMR spectroscopy. Among the most distinctive signals in the ^1^H-NMR spectrum of the P(DEGMA-*co*-OEGMA) macro-RAFT agent (P2 in Figure 1) is the signal at 4.1 ppm corresponding to the methylene protons “c” attached to the methacrylate in the OEGMA units; the methylenes “d” of the DEG units in the range from 3.75 to 3.55 ppm and the double signal (e) at 3.35 ppm corresponding to the terminal methyl proton “e” of both repetitive units. The ^1^H-NMR spectra of the CTA and of the other macro-CTA’s can be found in the Supplementary Information file (SI-file) (Figures S1 and S2).

Four P(DEGMA-*co*-OEGMA) macro-RAFT agents (first block) of different molar mass were prepared varying the monomer:CTA ratio, where the theoretical molar masswas estimated by using the ideal RAFT equation (Table 1), the molar composition of the copolymers was very similar to that initially utilized for the reaction due to the use of co-monomers with very similar reactivity towards radicals [31,32]. SEC analysis of these first blocks delivered experimental molar mass values close to the theoretical ones as well as low *Ð* values (1.05–1.10) (see Table 1), and monomodal and narrow elution time traces (see Figure S3 in SI-file) suggesting a good control of the RAFT polymerization. The actual copolymer composition of the obtained materials, as determined by ^1^H-NMR using the integration of signals “d” in the spectra and the equations reported in [30] for PDEGMA and POEGMA homopolymers, are also summarized in Table 1, whose composition values were close to the comonomer ratio (70:30) used in the feed.

### 3.2. Temperature-Responsive Behavior of P(DEGMA-co-OEGMA) Copolymers in Aqueous Media

**Figure 2:**
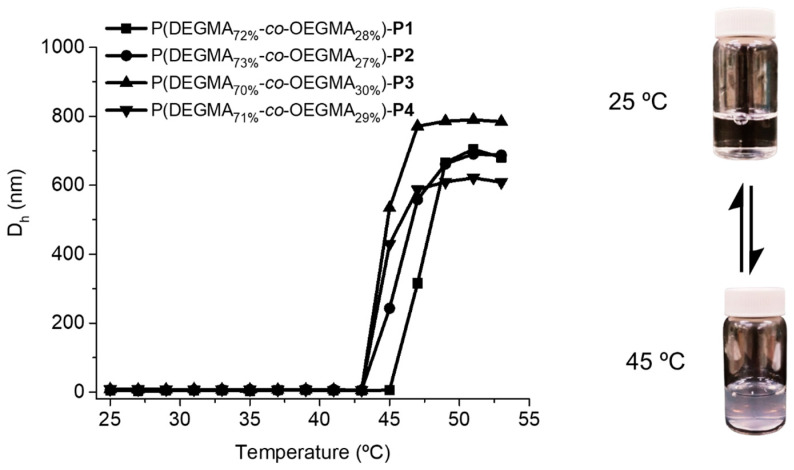
Analysis of *T_cp_* of aqueous solutions of P(DEGMA-*co*-OEGMA) copolymers (inserted pictures correspond to sample P(DEGMA_70%_-*co*-OEGMA_30%_), P3.

For the thermo-responsive studies, solutions were prepared by directly dissolving P(DEGMA-*co*-OEGMA) copolymers in deionized water (1 mg mL^−1^) at room temperature and subjected to DLS analysis. The cloud point (*T_cp_*) is a well-known parameter for the evaluation of thermo-responsiveness in polymer solutions. In our investigations, *T_cp_* was estimated by determining the temperature at which the *D*_h_ value experienced a sharp increase at a specific concentration [29]. The hydrophilic oligo(ethylene glycol) units may form hydrogen bonds with water molecules, while the backbones of the principal chains might undergo hydrophobic interactions. At the *T_cp_*_,_ the hydrogen bonds break, and the hydrophobic interactions start predominating, leading to the formation of aggregates and an increase in turbidity. On the other hand, it is well known that the *T_cp_* of aqueous dispersions containing DEGMA/OEGMA copolymers can be modulated in a wide temperature range [30,33]. Nonetheless, this behavior is not always straightforward to elucidate since combinations of OEGMA with a very similar monomer 2-hydroxyethyl methacrylate (HEMA) result in copolymers with either LCST or upper critical solution temperature (UCST) [34]. When the P(DEGMA-*co*-OEGMA) copolymer has a composition of around 70:30, the dispersion has a *T_cp_* value of 43 °C (as shown in Figure 2). However, the presence of salts influences the behavior of P(DEGMA-*co*-OEGMA) copolymers in aqueous media. Therefore, the *T_cp_* can also be displaced depending on the composition of the aqueous medium. For instance, *T_cp_* values of P(DEGMA-*co*-OEGMA) copolymers PBS buffer, are lower than in pure water [30,35].

Note that the *T_cp_* value of sample P1 is 45 °C (see Figure 2) and higher than that of the other three samples. This sample has the lowest molar mass, 11,700 g mol^−1^; half of that of sample P2. The effect of higher LCST for lower molar mass samples of the same polymer is reported, for instance, for the cases of poly(*N*-vinylcaprolactam) [36] and poly(2-dimethyl(aminoethyl) methacrylate) aqueous solutions [37].

### 3.3. Synthesis and Characterization of P(DEGMA-co-OEGMA)-b-PNIPAAm and P(DEGMA-co-OEGMA)-b-P(NIPAAm-co-BA)

**Scheme 3:**
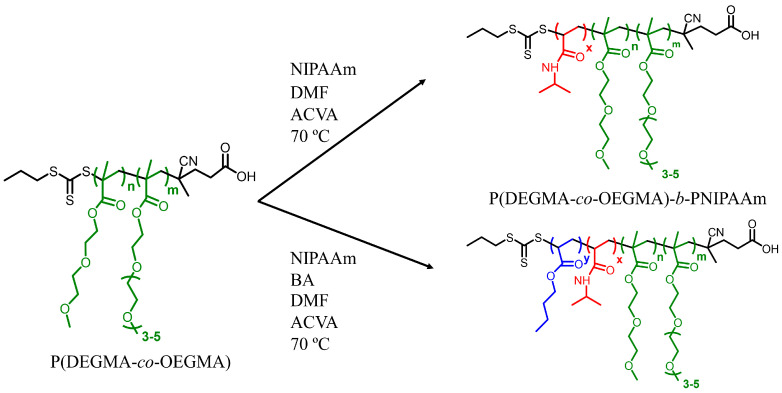
Schematic representation of the synthesis route of P(DEGMA-*co*-OEGMA)-*b*-PNIPAAm and P(DEGMA-*co*-OEGMA)-*b*-P(NIPAAm-*co*-BA) block copolymers via RAFT polymerization. Colors of polymeric structures correspond to Scheme 1 and Figure 9c.

Double thermo-responsive P(DEGMA-*co*-OEGMA)-*b*-PNIPAAm block copolymers were prepared using P(DEGMA-*co*-OEGMA) as a macro-RAFT agent (see Section 2 for details). To lower the *T_cp_* value of the PNIPAAm segment in the corresponding aqueous solution, 10 mol% of BA was incorporated as a co-monomer during the RAFT synthesis of the second block (see Scheme 3). The aim was to obtain self-assembled core–shell structures at 25 °C in aqueous media featuring a hydrophilic P(DEGMA-*co*-OEGMA) shell and a hydrophobic P(NIPAAm-*co*-BA) core.

**Figure 3:**
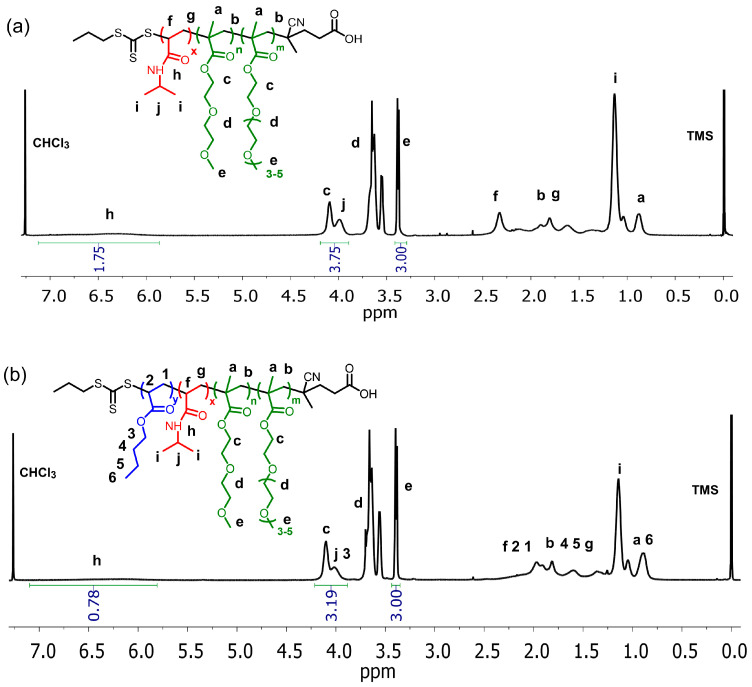
^1^H-NMR spectra (400 MHz) in CDCl_3_ of block copolymers: (**a**) P(DEGMA-*co*-OEGMA)-*b*-PNIPAAm (P2-2), (**b**) P(DEGMA-*co*-OEGMA)-*b*-P(NIPAAm-*co*-BA) (P2-3). Colors of polymeric structures correspond to Scheme 1 and Figure 9c.

Figure 3 displays the ^1^H-NMR spectra of the P(DEGMA-*co*-OEGMA)-*b*-PNIPAAm sample P2-2 and of the P(DEGMA-*co*-OEGMA)-*b*-P(NIPAAm-*co*-BA) sample P2-3. A part from the same P(DEGMA-*co*-OEGMA) signals observed at the same chemical shift values as those found in Figure 2, Figure 3a,b also show new signals ascribed to the PNIPAAm and the P(NIPAAm-*co*-BA) segments, respectively. The most prominent new signals are: the methine proton of the isopropyl groups of PNIPAAm observed at 3.97 ppm, which overlaps with the methylene protons (2) of PBA; a broad signal between 5.7 and 7.1 ppm attributed to the amide proton (h) of PNIPAAm; and, at 1.13 ppm, the methyl protons (i) assigned to the isopropyl groups of PNIPAAm. The methyl protons of the PBA units overlap with the methyl groups of the methacrylate moieties of the P(DEGMA-*co*-OEGMA) block [38]. The molar ratio of block copolymers was determined by comparing the integral value of signal (e) for the P(DEGMA-*co*-OEGMA) block to that of signal (h) of PNIPAAm. In the case of the segments containing PBA, the molar ratio of PBA to PNIPAAm was determined by comparing the integral value of signal (h) to the integral value between 3.8 and 4.25 ppm (signals c, j, and 3). The ^1^H-NMR spectra of the other prepared block copolymers can be found in the SI file (Figure S4).

A series of block copolymers featuring different molar mass was prepared by varying NIPAAm:BA:macro-CTA ratio; the corresponding theoretical molar masses were estimated using an adaptation of the ideal RAFT equation, which considers the *M*_n_ of the respective P(DEGMA-*co*-OEGMA) macro-RAFT agent. Table 2 summarizes the results of these copolymerization reactions, where it can be observed that the experimental molar masses obtained were in proper agreement with the theoretical values due to the similar reactivity ratios of NIPAAm and BA; thus, a statistical copolymer block would be expected [39]. In general, the measured *Ð* values were below 1.13, suggesting proper control during the RAFT polymerizations. The obtained block copolymers featured a monomodal, narrow, and well-defined molar mass distribution (see Table 2 and Figure S5 in SI-file).

The thermal properties of the synthesized copolymers were examined via DSC in the temperature range from −80 to 200 °C. The glass transition temperature (*T*_g_) of linear PNIPAAm can be found between 85 and 130 °C depending on its molar mass [40], whereas it has been reported that the *T*_g_ of P(DEGMA-*co*-OEGMA) copolymers can be adjusted between −53 and −59 °C with the co-monomer composition [35]. The DSC investigations of the P(DEGMA-*co*-OEGMA)_37%_-*b*-PNIPAAm_63%_ and P(DEGMA-*co*-OEGMA)_48%_-*b*-P(NIPAAm_43%_-*co*-BA_9%_) block copolymers yielded two glass transitions due to phase separation between the different segments of the block copolymers. The *T*_g_’s of PNIPAAm and P(DEGMA-*co*-OEGMA) segments were observed at 73 and −45 °C, respectively, and that of PNIPAAm shifted to lower values when BA units were incorporated as comonomer (see Figure S6 in the SI-file). The experimentally determined two *T*_g_’s are, to a large extent, comparable to those of the respective (homo-) co-polymer segments, which confirms the microphase-separation of the block segments further indicating the successful formation of block copolymer materials. The recorded thermograms of the other investigated block copolymers can also be found in the SI-file (Figures S7 and S8).

### 3.4. Thermo-Responsive Behavior of P(DEGMA-co-OEGMA)-b-PNIPAAm and P(DEGMA-co-OEGMA)-b-P(NIPAAm-co-BA) Block Copolymers in Aqueous Media

**Figure 4:**
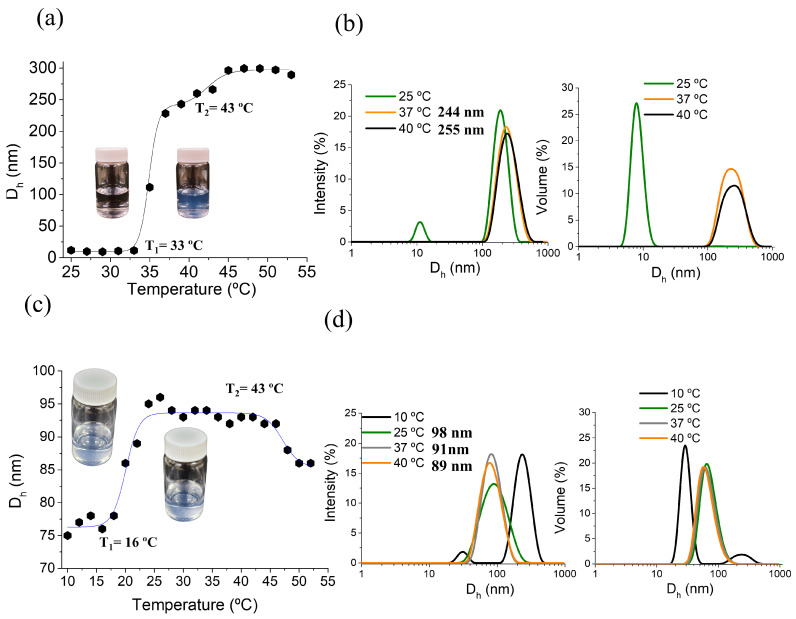
Analysis of size change in aqueous media and size distribution at different temperatures for block copolymers (1 mg mL^−1^): (**a**,**b**) P(DEGMA-*co*-OEGMA)_37%_-*b*-PNIPAAm_63%_; (**c**,**d**) P(DEGMA-*co*-OEGMA)_48%_-*b*-P(NIPAAm_43%_-*co*-BA_9%_).

The self-assembly behavior of the block copolymers against temperature in aqueous media was studied by monitoring their aggregation via DLS. P(DEGMA-*co*-OEGMA)-*b*-PNIPAAm block copolymers were directly dissolved in deionized water (1 mg mL^−1^), while the P(DEGMA-*co*-OEGMA)-*b*-P(NIPAAm-*co*-BA) block copolymers were first dissolved in DMF and slowly dripped into deionized water followed by dialysis for 24 h to remove DMF; the final concentration was adjusted to a value of 1 mg mL^−1^. Figure 4 shows the temperature-induced self-assembly behavior of samples P(DEGMA-*co*-OEGMA)_37%_-*b*-PNIPAAm_63%_ and P(DEGMA-*co*-OEGMA)_48%_-*b*-P(NIPAAm_43%_-*co*-BA_9%_) (subscript numbers indicate molar fractions in the block copolymers) in terms of DLS measurements.

Figure 4a shows a two-step increase in particle size with a first increase in *D*_h_ at a temperature close to the LCST of PNIPAAm (33 °C) suggesting the formation of aggregates. When temperature further increases above this latter value, the PNIPAAm segments are dehydrated, and the dispersion undergoes a self-assembly process to form aggregates consisting presumably of an insoluble PNIPAAm core surrounded by a corona of the solvated segments of the P(DEGMA-*co*-OEGMA) [19]. When temperature further increases above the *T*_cp_ of the P(DEGMA-*co*-OEGMA) block (whose chains turn insoluble and accumulate on the surface of the PNIPAAm core), it leads to the formation of larger aggregates as evidenced by a further increase in *D*_h_ starting at 43 °C; these experimental values were also fit to double-Boltzmann model (Figure 4a). Both blocks solubilize again when the temperature gradually decreases to room temperature. The particle size distribution at three different temperatures is also displayed in Figure 4b. The size distributions, as obtained via DLS, are plotted by intensity and by volume. When the block copolymers form uniform aggregates, both distributions, by intensity and by volume, are similar. However, when aggregates disassemble at room temperature (25 °C), two distributions of sizes can be observed presumably corresponding to single polymer chains and remaining aggregates. The size distribution by volume might be a better representation of the relative number of single chains as compared to that of aggregates (Figure 4b, right plot) because the size distribution by intensity overestimates the number of aggregates due to the strong dependence of scattering intensity on particle size [41] (Figure 4b, middle plot).

In the case of the double thermo-responsive block copolymers containing hydrophobic BA units (P(DEGMA-*co*-OEGMA)_48%_-*b*-P(NIPAAm_43%_-*co*-BA_9%_)), Figure 4c shows that relatively stable aggregates are formed below 16 °C. Presumably, these are also core–shell structures; however, they aggregate at room temperature, and their stability above 27 °C results in a slight shrinkage of aggregates upon heating (without further aggregation) until the *T_cp_* of the P(DEGMA-*co*-OEGMA) blocks is reached (43 °C). Further heating to temperatures higher than 50 °C leads to stronger shrinkage and finally to precipitation; this same behavior has been reported for double thermo-responsive PNIPAM-*b*-PDEMAEMA-*b*-PS triblock copolymers [42]. Adding hydrophobic units into thermo-responsive polymers shifts their corresponding LCST to lower values; for example, the *T_cp_* of PNIPAAm can be shifted between 10–16 °C depending on the copolymer composition. This phenomenon has already been reported for hydrophobic BA units contributing to the formation of polymer aggregates that self-assemble at 25 °C featuring monomodal size distributions (with a particle size of ca. 89 nm) [38]. However, when the temperature decreases to 10 °C, the polymer aggregates dissociate leading to the rise in two size distributions featuring small and larger aggregates (Figure 4d, middle plot). The relative amount of small and large aggregates can be assessed with the aid of the corresponding size distribution by volume, which shows that small aggregates are preferably formed (Figure 4d, right plot). It can be summarized that heating from room temperature up to the physiological relevant temperature of 37 °C, the investigated P(DEGMA-*co*-OEGMA)-*b*-PNIPAAm block copolymers self-assemble into nanostructures of ca. ~200 nm, while the investigated P(DEGMA-*co*-OEGMA)-*b*-P(NIPAAm-*co*-BA) block copolymers shrink to form stable nanostructures in the range of ca. ~95 nm. Upon cooling from room temperature, the investigated P(DEGMA-*co*-OEGMA)-*b*-PNIPAAm block copolymers dissociate to single block copolymer chains of ca. ~8 nm, while the investigated P(DEGMA-*co*-OEGMA)-*b*-P(NIPAAm-*co*-BA) block copolymers form more persistent aggregates of relatively small size (ca. ~30 nm). The SI-file shows the thermo-responsive behavior of all the investigated block copolymers (Figures S9 and S10).

### 3.5. Aminolysis of Trithiocarbonate End-Groups of Block Copolymers to Form Block Copolymers with Thiol End-Groups

**Scheme 4:**
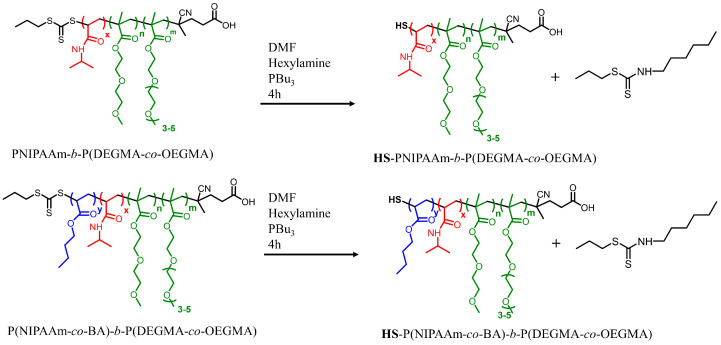
Schematic representation of the preparation of thiol-terminated block copolymers via aminolysis of trithiocarbonates. Colors of polymeric structures correspond to Scheme 1 and Figure 9c.

The preparation of reactive thiol-terminated polymers using aminolysis reaction as a post-functionalization strategy is of relevance in polymer synthesis as these materials can be used in different applications including GNRDs stabilizers [12]. However, it is important to avoid the formation of undesired disulfide bonds between two polymer chain ends. Among the contributing factors are the chemical structure of the corresponding (co)polymer, the reaction time and solvent, the type of amine used, and/or the presence of oxygen [15,43,44]. It has been reported that the presence of phosphine during the modification decreases the coupling between polymer chains as a consequence of the formation of disulfide bonds [8] (see Scheme 4).

Copolymers prepared via RAFT polymerization exhibit a strong absorption band at 310 nm due to the presence of a terminal trithiocarbonate group featuring a n-π* transition [44]. Table 3 shows a list of the block copolymers selected for post-functionalization based on their molar mass, self-assembly behavior, and size distribution.

**Figure 5:**
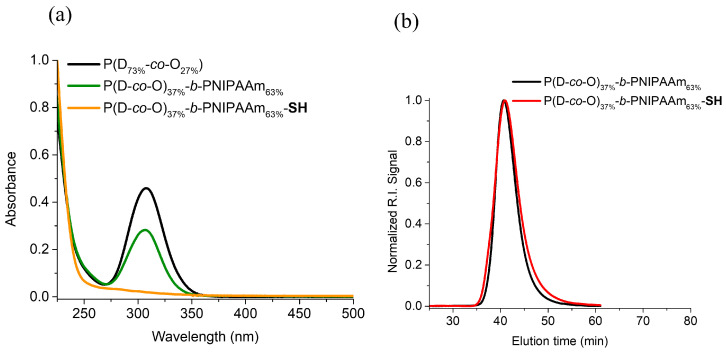
(**a**) UV-vis spectra (c = 1 mg mL^−1^ in ethanol), and (**b**) SEC traces in DMF for P(DEGMA-*co*-OEGMA)_37%_-*b*-PNIPAAm_63%_ (*M*_n_ = 32,600 g mol^−1^, *Ð* = 1.09) and P(DEGMA-*co*-OEGMA)_37%_-*b*-PNIPAAm_63%_-SH (*M*_n_ = 35,800 g mol^−1^, *Ð* = 1.11).

Representative results obtained from the aminolysis reactions of the investigated copolymers are displayed in Figure 5a (i.e., UV-vis spectra). The first product of the performed RAFT polymerizations, namely P(DEGMA_73%_-*co*-OEGMA_27%_) macro-CTA, exhibited a strong absorption band, whose intensity decreased as the chain-extension polymerization was performed (i.e., synthesis of the PNIPAAm block). After aminolysis of the trithiocarbonate end-groups, the absorption band vanished indicating the formation of thiol-terminated polymers.

Figure 5b displays the corresponding SEC traces of P(DEGMA-*co*-OEGMA)_37%_-*b*-PNIPAAm_63%_ with trithiocarbonate end-group and with thiol end-group. The elution time trace ascribed to the thiol-terminated copolymer practically remains unaltered as compared to the trace of its precursor material. Nevertheless, a slight broadening can be observed, indicating a slight change in the molar mass and dispersity of the copolymer. The UV-Vis spectra of all the modified block copolymers can be found in the SI-file (Figure S11).

### 3.6. Preparation of Double Thermo-Responsive Copolymer@GNRDs Nanohybrids: Self-Assembly and Stability Investigations

**Figure 6:**
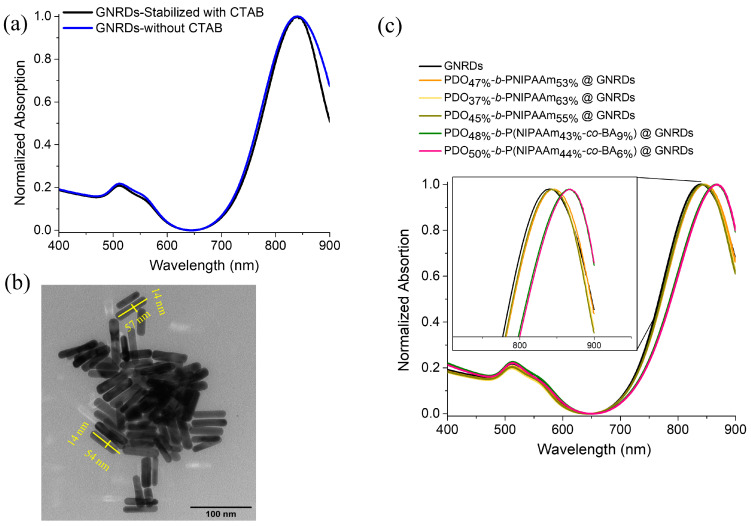
Characterization of the synthesized GNRDs featuring an aspect ratio of 3.98: (**a**) UV-Vis spectra with and without CTAB; (**b**) TEM micrograph of GNRDs dispersed after removing CTAB; (**c**) UV-Vis spectra of the block copolymer@GNRDs nanohybrids in an aqueous medium.

GNRDs were synthesized via a seed-mediated growth method [26,27]. Figure 6a displays the UV-vis spectra of the GNRDs, where the extinction spectra of the GNRDs in water exhibit two absorption bands at λ_max_ = 510 nm corresponding to the transverse surface plasmon resonance in the visible range, and the longitudinal surface plasmon resonance (LSPR) in the near-infrared (NIR) range at ca. λ_max_ = 820 nm. It can also be observed that the LSPR is slightly modified as the CTAB stabilizer is removed from the system. The LSPR in the NIR region is particularly relevant for this current investigation as well as the relationship of its intensity and position with the surface modification. Figure 6b shows a TEM micrograph featuring well-defined rods, whose average dimensions were 56.18 ± 5.38 nm in length and 14.08 ± 1.40 nm in width yielding an aspect ratio (AR) of 3.98 (length/diameter).

After synthesis and purification, the GNRDs were further modified with the previously prepared thiol end-functionalized block copolymers. A series of P(DEGMA-*co*-OEGMA)-*b*-PNIPAAm-SH and P(DEGMA-*co*-OEGMA)-*b*-P(NIPAAm-*co*-BA)-SH block copolymers were immobilized onto the surface of GNRDs via a “grafting to” approach. The aim of this was to protect GNRDs from self-aggregation and provide them with a thermo-responsive aggregation/disaggregation property by employing the proposed block copolymers. The grafting reaction was carried out in an aqueous medium at room temperature using a simple mixing. Figure 6c shows that the LSPR band presented a slight shift upon polymer coating. This behavior is more evident in the case of P(DEGMA-*co*-OEGMA)-*b*-P(NIPAAm-*co*-BA)@GNRDs nanohybrids presumably because they exhibit more hydrophobic characteristics. This shift in the LSPR band might be due to changes in the local dielectric coefficient and surface refractive index [45]. It is also evident that there is no broadening of the LSPR band, which suggests the absence of GNRDs aggregation after modification with the block copolymers.

**Figure 7:**
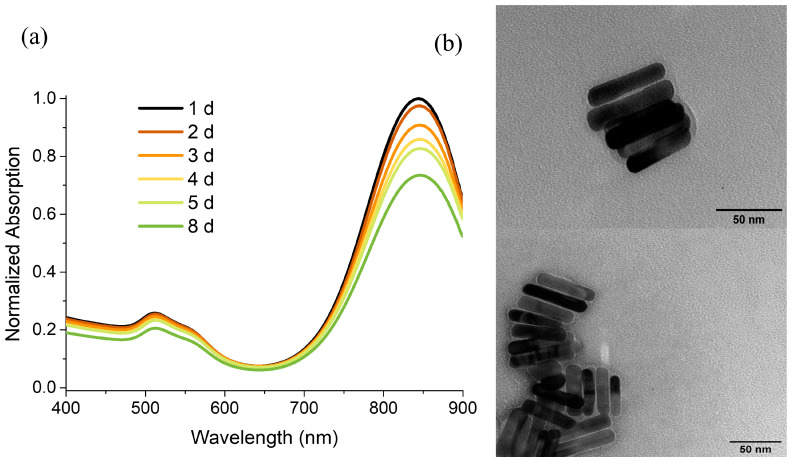
Characterization of P(EGMA-*co*-OEGMA)_37%_-*b*-PNIPAAm_63%_@GNRDs nanohybrid: (**a**) UV-Vis spectra of dispersions monitored for 8 days at room temperature, (**b**) Representative TEM micrographs of block copolymer@GNRDs in dry state (as stained with uranyl acetate).

On the other hand, it is also relevant for biomedical applications to investigate the stability of the nanohybrids in dispersion over time, which can be monitored by assessing possible changes in the UV-Vis absorption spectra of the nanohybrids. Thus, this optical stability was examined for 8 days via the analysis of the position and broadness of the LSPR band at 25 °C. Figure 7a shows that the LSPR band did not present any significant displacement to higher wavelengths or change in broadness during the investigated period of time. Although the intensity of the absorbance band decreases over time, the dispersions did not show any precipitation at room temperature (see Figure S12 in the SI file). Meanwhile, TEM micrographs of P(DEGMA-*co*-OEGMA)_37%_-*b*-PNIPAAm_63%_@GNRDs dried at room temperature are displayed in Figure 7b, in which the negatively stained uranyl acetate polymer shell can be observed as a halo featuring the copolymer coating (See Figure S13 in SI file for additional micrographs).

### 3.7. Behavior of Copolymer@GNRDs Nanohybrids upon Heating and by NIR-Irradiation

The block copolymer@GNRDs nanohybrids are expected to retain the thermo-responsiveness of P(DEGMA-*co*-OEGMA)-*b*-PNIPAAm and P(DEGMA-*co*-OEGMA)-*b*-P(NIPAAm-*co*-BA) block copolymers [15,26,46].

**Figure 8:**
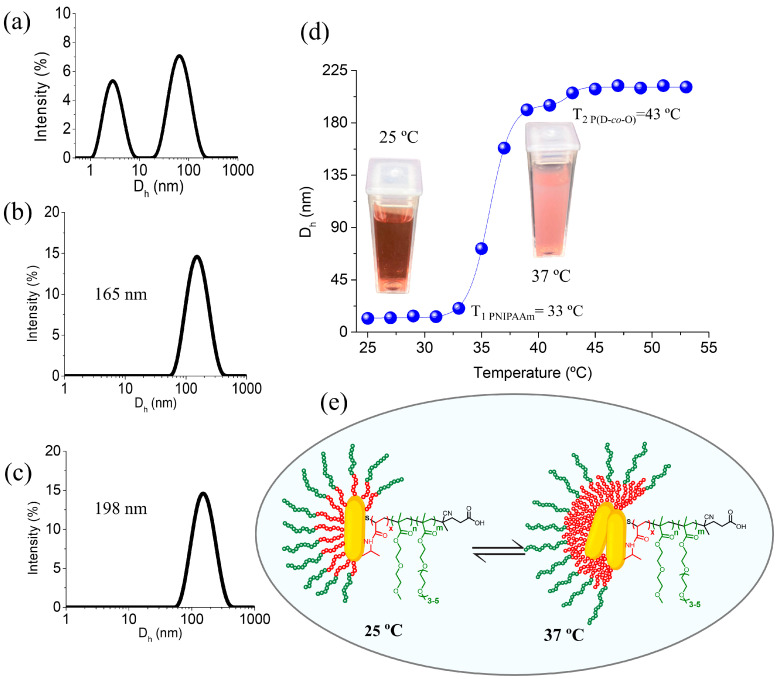
Size distributions of P(EGMA-*co*-OEGMA)_37%_-*b*-PNIPAAm_63%_@GNRDs nanohybrids in an aqueous medium at different temperatures: (**a**) 25 °C; (**b**) 27 °C; (**c**) 40 °C; (**d**) self-assembly behavior of the nanohybrid in an aqueous medium upon increasing temperature; (**e**) schematic representation of the self-assembly behavior. Colors of polymeric structures correspond to Scheme 1.

Changes in temperature of the block copolymer@GNRDs nanohybrids in aqueous suspension were probed using DLS. It is known that for rod-like particles, the estimation of sizes in terms of the diffusion coefficients produces bimodal distributions (Figure 8a), whose peaks do not correspond to either the longitudinal or transverse dimensions of the rods [45]. The small size peak is attributed to the rotational diffusion coefficient of the rods and the large size peak simply means that the nanorods have the same diffusion coefficient as a spherical gold nanoparticle of that size. Nevertheless, the bimodal distribution can be taken as an indication of the presence of rod-like particles in a sample. Such is the case observed for our block copolymer@GNRDs nanohybrids at 25 °C (Figure 8a). In this situation, the P(DEGMA-*co*-OEGMA)-*b*-PNIPAAm polymeric chains are hydrated, forming a solvated polymer brush-type coating on the GNRDs.

At higher temperatures, on the other hand, the suspensions are expected to form aggregates with shapes that are closer to that of a sphere, leading to monomodal distributions in the DLS estimations (Figure 8b,c); in such cases, the estimated sizes are expected to be more reliable. Thus, changes from bimodal to a monomodal distribution in the DLS estimations were taken as a signature of aggregation, and the peaks of the monomodal distributions were used to estimate the aggregate sizes.

We observe that at 37 °C the DLS estimation exhibits a monomodal size distribution with a maximum at *D_h_* =165 nm. The increase in particle size can be explained by the dehydration of the PNIPAAm block above its *T_cp_*, which forms a semispherical aggregate with a hydrophobic core of PNIPAAm@GNRDs and a P(DEGMA-*co*-OEGMA) hydrophilic shell (Figure 8b). Increasing the temperature further to 40 °C, which is close to the *T_cp_* of the P(DEGMA-*co*-OEGMA) thermo-responsive block (Figure 8c), increases the estimated D_h_ of the aggregates to 198 nm. Figure 8d,e illustrate the self-assembly behavior of the P(EGMA-*co*-OEGMA)_37%-_*b*_-_PNIPAAm_63%_@GNRDs in aqueous suspension. An increase in the turbidity of the suspension due to the aggregation of the copolymer coating can be clearly observed in the photographs, but it is important to mention that these dispersions did not show any precipitation until 50 °C. More information on this is presented in Table S1 of the SI-file.

**Figure 9:**
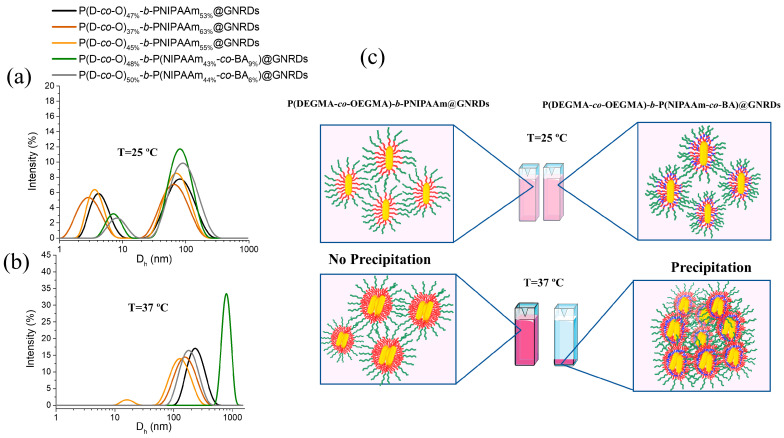
Size distributions of block copolymers@GNRDs nanohybrids: (**a**) at 25 °C; (**b**) at 37 °C; (**c**) schematic representation of the behavior of the block copolymer@GNRDs nanohybrids in the temperature range between 25 and 37 °C.

The molar mass of the P(DEGMA-*co*-OEGMA) segment has an important effect on the stabilization of the nanohybrids at temperatures above the *T_cp_* of the PNIPAAm segment as it contributes to preserving the stability of the hybrid aggregates featuring *D_h_* values <200 nm at 37 °C (see Figure 9b for the cases P(EGMA-*co*-OEGMA)_37%_-*b*-PNIPAAm_63%_@GNRDs and P(EGMA-*co*-OEGMA)_45%_-*b*-PNIPAAm_55%_@GNRDs).

However, for the P(DEGMA-*co*-OEGMA)-*b*-P(NIPAAm-*co*-BA)@GNRDs suspensions (containing hydrophobic BA units), the thermo-responsiveness derived from the PNIPAAm copolymer segment is already active at 16 °C leading to the formation of stable aggregates at room temperature, and heating up to 37 °C increases hydrophobic polymer-polymer-GNRDS interactions leading to the destabilization of nanohybrids aggregates and to partial precipitation when the BA content is relatively high (9 mol%), but not when is lower (6 mol%). The fact that there are two apparent size distributions of nanohybrids at room temperature (Figure 9a) may be ascribed to the anisotropy of the block copolymer-coated GNRDs as already discussed above [47]. A schematic representation describing this hypothesized temperature-driven self-assembly behavior at 25 and 37 °C is displayed in Figure 9c. Additional DLS results for measurements performed at 37 °C can be found in Table S2 in the SI-file. Considering that after activating the thermo-responsive behavior the self-aggregation phenomena upon heating lead ultimately to a phase separation, it is not common to find in the literature, a report on the self-aggregation behavior of nanohybrids above their aggregation temperature. This current investigation uses block copolymers featuring a double thermo-responsiveness hypothesizing that between both transition temperatures, there may be a sweet spot where such nanohybrids remain stable.

**Figure 10:**
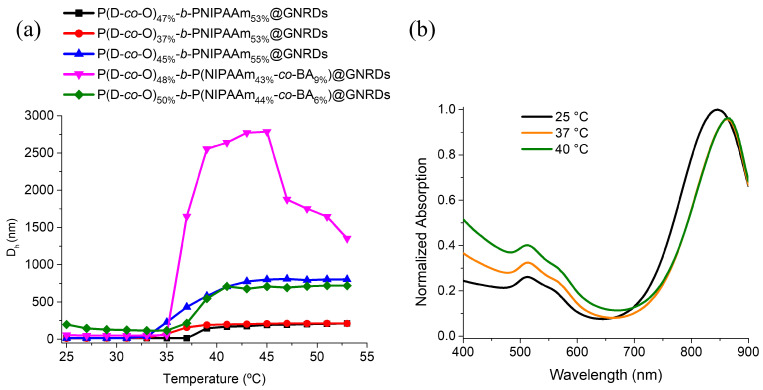
(**a**) Evolution of size vs. temperature for different block copolymer@GNRDs nanohybrids in an aqueous medium; (**b**) UV-Vis absorption spectra of P(EGMA-*co*-OEGMA)_37%_-*b*-PNIPAAm_63%_@GNRDs in an aqueous medium at 25, 37 and 40 °C.

In our case, the *D_h_* of the nanohybrids can be maintained below 200 nm at 37 °C via the manipulation of various contributing factors such as the molar mass of the block copolymers and/or their copolymer composition (Figure 10a). In the case of P(DEGMA-*co*-OEGMA)-*b*-PNIPAAm, the second transition temperature ascribed to the P(DEGMA-*co*-OEGMA) block does not lead to a change in the size of the nanohybrids when the copolymer chains are attached to the GNRDs. However, in the case of the more hydrophobic copolymers containing BA units, this second transition temperature shifts to lower values and provokes further aggregation and even precipitation of the entire nanohybrids (i.e., when the copolymer chains are attached to GNRDs) (Figure 10a). On the other hand, it is also relevant to investigate how the LSPR band changes as the block copolymer self-assembles on the GNRDs surface upon increasing temperature. Upon heating the block copolymer@GNRDs dispersion above the *T_cp_* of the PNIPAAm segment, the latter undergoes a phase transition and precipitates over the GNRDs surface. This change in aqueous medium leads to an increase in the refractive index of the dispersion, which in turn causes a shift in the LSPR band. A representative behavior of the extinction spectra of P(DEGMA-*co*-OEGMA)_37%_-*b*-PNIPAAm_63%_@GNRDs in the temperature range from 25 up to 40 °C is shown in Figure 10b.

A shift in the LSPR was observed with increasing temperature, this effect is not different at 40 °C as the *T_cp_* of the first block P(DEGMA-*co*-OEGMA) is still not reached and the aggregates remain stable. The situation changes when the polymer interactions increase causing precipitation of the samples that contain BA-units. UV-Vis spectra of the other nanohybrids can be found in the SI file (Figure S14).

**Figure 11:**
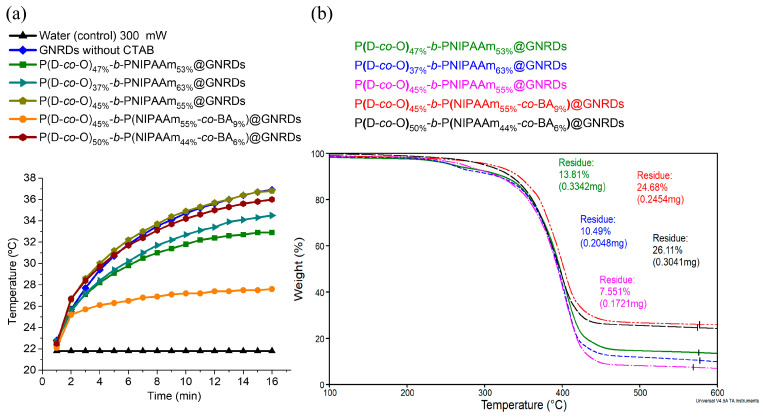
(**a**) Surrounding temperature of block copolymer@GNRDs and GNRDs aqueous dispersions vs. NIR-irradiation time (at 795 nm, 300 mW); (**b**) TGA thermograms of the investigated block copolymer@GNRDs.

The photo-thermal behavior of the P(DEGMA-*co*-OEGMA)-*b*-PNIPAAm@GNRDs and P(DEGMA-*co*-OEGMA)-*b*-P(NIPAAm-*co*-BA)@GNRDs nanohybrids in an aqueous medium was investigated with the aid of a 785 nm NIR laser. The measurements consisted of irradiating the sample (2 mL) for 16 min with a laser output power of 300 mW while recording the temperature of the system every 1 s. Pristine distilled water was irradiated at 300 mW as a control experiment to ensure that the temperature evolution recorded in the case of nanohybrid dispersion was not due to energy absorption of water or of the measuring instrument (see Figure 11a). The GNRDs stabilized without CTAB dispersed in an aqueous medium showed a temperature increment of 16 °C after the experiment. For the block copolymer@GNRDs nanohybrid dispersions, the temperature rise was slightly lower than that recorded for GNRDs presumably due to the shift in the LSPR band and the change in the refractive index of the dispersion as the transition temperature of the copolymer is reached. For example, a temperature increment of 12 °C was observed for the P(EGMA-*co*-OEGMA)_47%_-*b*-PNIPAAm_53%_@GNRDs system as well as an increase in turbidity of the suspension during the NIR-irradiation. However, this temperature increase is still considerable and may be suitable for photo-thermal therapies. Only in the case of the already detected and discussed P(DEGMA-*co*-OEGMA)_45%_-*b*-P(NIPAAm_55%_-*co*-BA_9%_)@GNRDs system featuring a significant precipitation of the nanohybrid, the heating of the surrounding is not sufficient for the requirements of the intended application. Figure 11b reveals the content of GNRDs in the nanohybrid systems as estimated via TGA. For this purpose, the wt.% residue of each block copolymer@GNRDs system was determined at 600 °C and the obtained wt.% residue values ranged between 8 and 26 wt.% depending on the preparation method and copolymer material utilized. The content of the GNRDs was estimated by the difference in the residue values of the block copolymer with and without GNRDs measured at the same temperature (600 °C). However, the GNRD content does not seem to influence the measured temperature increase provoked by NIR-irradiation. The onset of the decomposition step of the block copolymers can be observed at ca. 350 °C for all the investigated cases. TGA thermograms of block copolymers without GNRDs can be found in Figure S15 (SI-file).

## 4. Conclusions

In this contribution, double thermo-responsive block copolymers of different compositions and molar mass were synthesized via RAFT copolymerization using a trithiocarbonate CTA. The composition of the first block (composed of DEGMA and OEGMA units) was adjusted accordingly to feature a response temperature above 40 °C, while the second block (composed of NIPAAm) was varied to show a response temperature above room temperature, but below the physiologically relevant temperature of 37 °C. In a similar case to the latter, a transition temperature below room temperature was also achieved via the incorporation of BA units into the NIPAAm block.

Thiol-terminated block copolymers were prepared via aminolysis of the trithiocarbonate end-groups. Thereafter, the thiol-reactive groups were used for grafting the double thermo-responsive block copolymers onto GNRDs. The GNRDs grafted with block copolymers, where the second block is composed of NIPAAm units, remained stable in an aqueous dispersion for at least 8 days at room temperature; while at temperatures above their first response temperature, e.g., at 37 °C, the nanohybrids self-assemble to form aggregates with a *D_h_* < 200 nm without precipitating. In contrast, GNRDs grafted with block copolymers, where the second block is composed of NIPAM and BA units, already self-assembled at room temperature as the first response temperature is at 16 °C. At 37 °C, these nanohybrids show agglomeration between polymer aggregates leading to a partial precipitation, which strongly depends on the BA content (e.g., 9 mol% led to precipitation while 6 mol% did not) suggesting that the second transition temperature shifts to lower values. The photo-thermal effect remained active when the investigated block copolymers@GNRDs nanohybrids were irradiated in the NIR region. After 16 min of NIR-irradiation (795 nm, 300 mW), the nanohybrids showed a temperature increment of the systems in the range from 12 to 16 °C at a given concentration (except for the system containing 9 mol % of BA, which precipitated). All in all, the proposed block copolymer@GNRDs nanohybrids with two-temperature responses may be interesting candidates for a temperature-specific drug loading at their first response temperature and a specific release at their second response temperature under NIR-irradiation, which may open new possibilities for photo-thermal therapies.

## Figures and Tables

**Table 1:** Conditions for RAFT polymerization of P(DEGMA-*co*-OEGMA) in DMF at 70 °C for 4 h in the presence of a trithiocarbonate CTA.

Entry	Sample ^1^	Feed Molar Ratio [M]:[CTA]:[I]	P(D:O) ^2^ (mol%)	Conv. ^3^ (%)	*M_n_* Theo ^4^ (g/mol)	*M_n_* SEC ^5^ (g/mol)	*Ð* ^5^ M_w_/M_n_
P1	P(DEGMA_72%_-*co*-OEGMA_28%_)	[66]:[1]:[0.1]	72:28	76	11,700	11,100	1.10
P2	P(DEGMA_73%_-*co*-OEGMA_27%_)	[134]:[1]:[0.1]	73:27	75	22,800	17,500	1.05
P3	P(DEGMA_70%_-*co*-OEGMA_30%_)	[200]:[1]:[0.1]	70:30	63	27,900	25,800	1.07
P4	P(DEGMA_71%_-*co*-OEGMA_29%_)	[270]:[1]:[0.1]	71:29	58	34,700	30,100	1.06

^1^ The percentage subscripts numbers represent the composition of the copolymers by ^1^H-NMR (400 MHz). ^2^ The composition was determined by ^1^H-NMR. ^3^ The conversion was determined by ^1^H-NMR. ^4^ The theoretical molecular weight was calculated by using the ideal RAFT equation: $M_{n,theo}=\left[\left(\frac{\left[Mon\right]}{\left[CTA\right]}\right)\cdot {Mon}_{w}\cdot Conv\right]+M_{n\;CTA}$, where [Mon] is the sum of [D] + [O], Mon_w_ is the molar mass of the monomer, in this case ${Mon}_{w}=[\left(0.7\;M_{w}DEGMA\right)+\left(0.3\;M_{w}OEGMA\right)]$ and M_wCTA_ is the molar mass of CTA. ^5^ Determined by SEC in DMF at 40 °C with RI and LS detectors using poly(ethylene oxide) linear standards for verification of the LS detector. P(DEGMA-*co*-OEGMA) *dn*/*dc* = 0.091 mL g^−1^.

**Table 2:** Experimental conditions and results of the RAFT polymerizations for the synthesis of P(DEGMA-*co*-OEGMA)-*b*-PNIPAAm and P(DEGMA-*co*-OEGMA)-*b*-P(NIPAAm-*co*-BA) in DMF at 70 °C for 2 h using P(DEGMA-*co*-OEGMA)s as macro-RAFT agents (mCTAs).

Entry	Sample ^1^	Feed Molar Ratio [Mon]: [mCTA]:[I] ^2^	Composition PDO:PNIPAAm:PBA ^3^ (mol %)	Conv. ^4^ (%)	*M*_n_ Theo ^5^ (g mol^−1^)	*M*_n_ SEC ^6^ (g mol^−1^)	*Ð* ^6^
P1	P(D_72%_-*co*-O_28%_)	------	100:0:0	76	11,700	11,100	1.10
P1-1	P(D-*co*-O)_68%_-*b*-PNIPAAm_32%_	[50]:[1]:[0.1]	68:32:0	58	14,500	17,500	1.05
P1-2	P(D-*co*-O)_47%_-*b*-PNIPAAm_53%_	[100]:[1]:[0.1]	47:53:0	71	20,100	18,900	1.04
P1-3	P(D-*co*-O)_49%_-*b*-P(NIPAAm_40%_-*co*-BA_11%_)	[100]:[1]:[0.1]	49:40:11	65	18,600	19,300	1.05
P2	P(D_73%_-*co*-O_27%_)	------	100:0:0	75	22,500	17,500	1.05
P2-1	P(D-*co*-O)_63%_-*b*-PNIPAAm_37%_	[100]:[1]:[0.1]	63:37:0	72	30,400	24,500	1.05
P2-2	P(D-*co*-O)_37%_-*b*-PNIPAAm_63%_	[200]:[1]:[0.1]	37:63:0	71	35,500	32,600	1.09
P2-3	P(D-*co*-O)_48%_-*b*-P(NIPAAm_43%_-*co*-BA_9%_)	[200]:[1]:[0.1]	48:43:9	62	31,700	27,900	1.10
P3	P(D_70%_-*co*-O_30%_)	------	100:0:0	63	27,900	25,800	1.07
P3-1	P(D-*co*-O)_68%_-*b*-PNIPAAm_32%_	[100]:[1]:[0.1]	68:32:0	75	34,300	31,100	1.05
P3-2	P(D-*co*-O)_45%_-*b*-PNIPAAm_55%_	[200]:[1]:[0.1]	45:55:0	75	42,700	36,200	1.13
P3-3	P(D-*co*-O)_50%_-*b*-P(NIPAAm_44%_-*co*-BA_6%_)	[200]:[1]:[0.1]	50:44:6	59	39,300	34,200	1.09

^1^ The percentage subscripts numbers represent the composition of the copolymers as determined via ^1^H-NMR (400 MHz). ^2^ The block copolymers P1-3, P2-3 and P3-3, 10 mol% of BA was added according to NIPAAm. ^3^ The composition was determined by ^1^H-NMR. ^4^ The conversion was determined by ^1^H-NMR for the P(DEGMA-*co*-OEGMA)-*b*-PNIPAAm and determined gravimetrically for the P(DEGMA-*co*-OEGMA)-*b*-P(NIPAAm-*co*-BA). ^5^ The theoretical molar mass was calculated by the equation: $M_{n,theo}=\left[\left(\frac{\left[Mon\right]}{\left[macroCTA\right]}\right)\cdot {Mon}_{w}\cdot Conv\right]+M_{n\;macroCTA}$, where [Mon] = [NIPAAm] + [BA], Mon_w_ is the molar mass of the corresponding monomers, in this case ${Mon}_{w}=\left[\left(X\;M_{w}NIPAAm\right)+\left(Y\;M_{w}BA\right)\right]\;\mathrm{and}\;X+Y=100\%$ and *M*_n_ macroCTA is the molar mass of macroCTA. ^6^ Determined by SEC in DMF at 40 °C with RI and LS detectors using poly(ethylene oxide) linear standards of narrow dispersity for verification of LS detector; *dn*/*dc* = 0.091X + 0.070Y (mL g^−1^), being X and Y de molar ratios of P(DEGMA-*co*-OEGMA) = X and PNIPAM + PBA = Y in the copolymer.

**Table 3:** Block copolymers selected for aminolysis and some of their properties.

Sample ^1^	*M*_n_ ^2^ SEC ^1^ (g mol^−1^)	*Ð* ^2^	*T_cp_* 1 ^3^ (°C)	*T_cp_* 2 ^3^ (°C)	*T*_g_ 1 ^4^ (°C)	*T*_g_ 2 ^4^ (°C)
P(D-*co*-O)_47%_-*b*-PNIPAAm_53%_	19,800	1.04	33	43	−40	79
P(D-*co*-O)_37%_-*b*-PNIPAAm_63%_	32,600	1.09	33	43	−45	74
P(D-*co*-O)_45%_-*b*-PNIPAAm_55%_	36,200	1.13	35	43	−43	73
P(D-*co*-O)_48%_-*b*-P(NIPAAm_43%_-*co*-BA_9%_)	27,900	1.10	16	43	−35	64
P(D-*co*-O)_50%_-*b*-P(NIPAAm_44%_-*co*-BA_6%_)	34,200	1.09	16	43	−30	58

^1^ Subscript numbers represent the composition of the copolymers in mol%. ^2^ Determined via SEC in DMF at 40 °C with RI and LS detector using poly(ethylene oxide) linear standard for verification of LS detector. ^3^ Determined via DLS in aqueous media (1 mg mL^−1^). ^4^ Determined via DSC.

## Data Availability

Data are available upon reasonable request.

## Supplementary Materials

The following supporting information can be downloaded at: https://www.mdpi.com/article/10.3390/polym16233293/s1, Figure S1: ^1^H-NMR spectrum (400 MHz) in CDCl_3_ of chain transfer agent (CTA); Figure S2: ^1^H-NMR spectra (400 MHz) in CDCl_3_ of the P(DEGMA-*co*-OEGMA) copolymers: (a) P1; (b) P3; (c) P4; Figure S3: Normalized SEC traces (RI detector) for P(DEGMA-*co*-OEGMA) macro-CTA´s; Figure S4: ^1^H-NMR spectra (400 MHz) in CDCl_3_ of the block copolymers: (a) P1-2; (b) P1-3; (c) P3-2; (d) P3-3; Figure S5: Normalized SEC traces (RI detector) in DMF for P(DEGMA-*co*-OEGMA)-*b*-PNIPAAm and P(DEGMA-*co*-OEGMA)-*b*-P(NIPAAm-*co*-BA) block copolymers; Figure S6: DSC thermograms of the P(DEGMA-*co*-OEGMA)-*b*-PNIPAAm (P2-2) and P(DEGMA-*co*-OEGMA)-*b*-P(NIPAAm-*co*-BA) (P2-3) block copolymers; Figure S7: DSC thermograms of the block copolymers; (a) P1-2, (b) P3-2; Figure S8: DSC thermograms of the block copolymer P3-3; Figure S9: Determination of T_cp_ of block copolymers (1 mg mL^−1^); a) P(DEGMA-*co*-OEGMA)_47%_-*b*-PNIPAAm_53%_, (b) P(DEGMA-*co*-OEGMA)_45%_-*b*-PNIPAAm_55%_; Figure S10: Determination of T_cp_ of block copolymers (1 mg mL-1); (c) P(DEGMA-*co*-OEGMA)_50%_-*b*-P(NIPAAm_44%_-*co*-BA_6%_), (d) P(DEGMA-*co*-OEGMA)_49%_-*b*-P(NIPAAm_40%_-*co*-BA_11%_); Figure S11: UV-Vis spectra (c = 1 mg mL-1 in ethanol) of the block copolymers and thiol-terminated block copolymers; Figure S12: Optical stability with time: (a) P(DEGMA-*co*-OEGMA)_47%_-*b*-PNIPAAm_53%_@GNRD; (b) P(DEGMA-*co*-OEGMA)_45%_-*b*-PNIPAAm_55%_@GNRDs; (c) P(DEGMA-*co*-OEGMA)_48%_-*b*-P(NIPAAm_43%_-*co*-BA_9%_)@GNRDs; (d) P(DEGMA-*co*-OEGMA)_50%_-*b*-P(NIPAAm_44%_-*co*-BA_6%_)@GNRDs; Figure S13: TEM micrographs of the nanohybrids: (a) P(DEGMA-*co*-OEGMA)_45%_-*b*-PNIPAAm_55%_@GNRDs; (b) P(DEGMA-*co*-OEGMA)_50%_-*b*-P(NIPAAm_44%_-*co*-BA_6%_)@GNRDs; Figure S14: UV-Vis absorption spectra at 25, 37 and 40 °C for nanohybrids: (a) P(DEGMA-*co*-OEGMA)_47%_-*b*-PNIPAAm_53%_@GNRDs, (b) P(DEGMA-*co*-OEGMA)_45%_-*b*-PNIPAAm_55%_@GNRDs; Figure S15: TGA-thermograms of the block copolymers; Table S1: Aggregation of sample P(DEGMA-*co*-OEGMA)_37%_-*b*-PNIPAAm_63%_@GNRDs at different temperatures; Table S2: Aggregation data of block copolymers@GNRDs at 37 °C.
