# Supplementary material for: Surface Modification of Gold Nanorods (GNRDs) Using Double Thermo-Responsive Block Copolymers: Evaluation of Self-Assembly and Stability of Nanohybrids
Source: Polymers (Basel). 2024 Nov 26;16(23):3293. doi: 10.3390/polym16233293 (PMC11644200; doi:10.3390/polym16233293)
Supplement: Supplementary file 1 [file polymers-16-03293-s001.zip › polymers-3301972-supplementary.pdf]

Surface Modification of Gold Nanorods (GNRDs) using Double Thermo-responsive Block Copolymers: Evaluation of Self-assembly and Stability of Nanohybrids

Jesús E. Márquez-Castro[1], Angel Licea-Claverie[1*], Carlos Guerrero-Sanchez[2,3*] and Eugenio R. Méndez[4]

[1] Centro de Graduados e Investigación en Química, Tecnológico Nacional de México/Instituto Tecnológico de Tijuana, Tijuana, Mexico.

[2] Laboratory of Organic and Macromolecular Chemistry (IOMC) Friedrich Schiller University Jena, Jena, Germany.

[3] Jena Center for Soft Matter (JCSM), Friedrich Schiller University Jena, Jena, Germany.

[4] División de Física Aplicada, Centro de Investigación Científica y Educación Superior de Ensenada, Ensenada, Mexico.

* Correspondence: carlos.guerrero.sanchez@uni-jena.de; aliceac@tectijuana.mx; Tel.: CGS +49-364-1948598; ALC +52-664-6234043

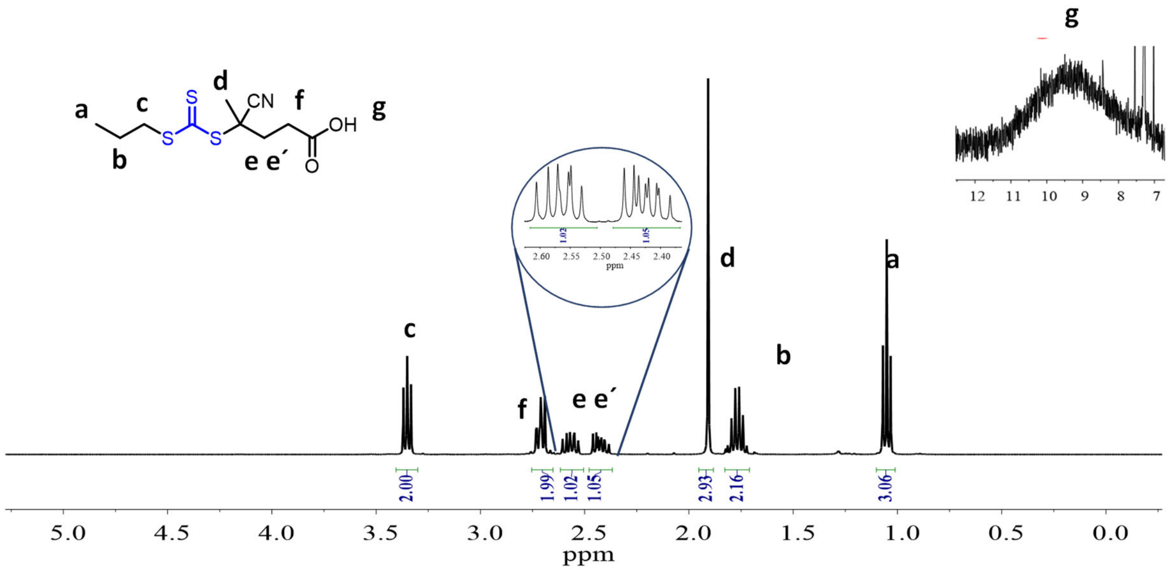

Figure S1. ^{1}H-NMR spectrum (400 MHz) in $CDCl_3$ of chain transfer agent (CTA).

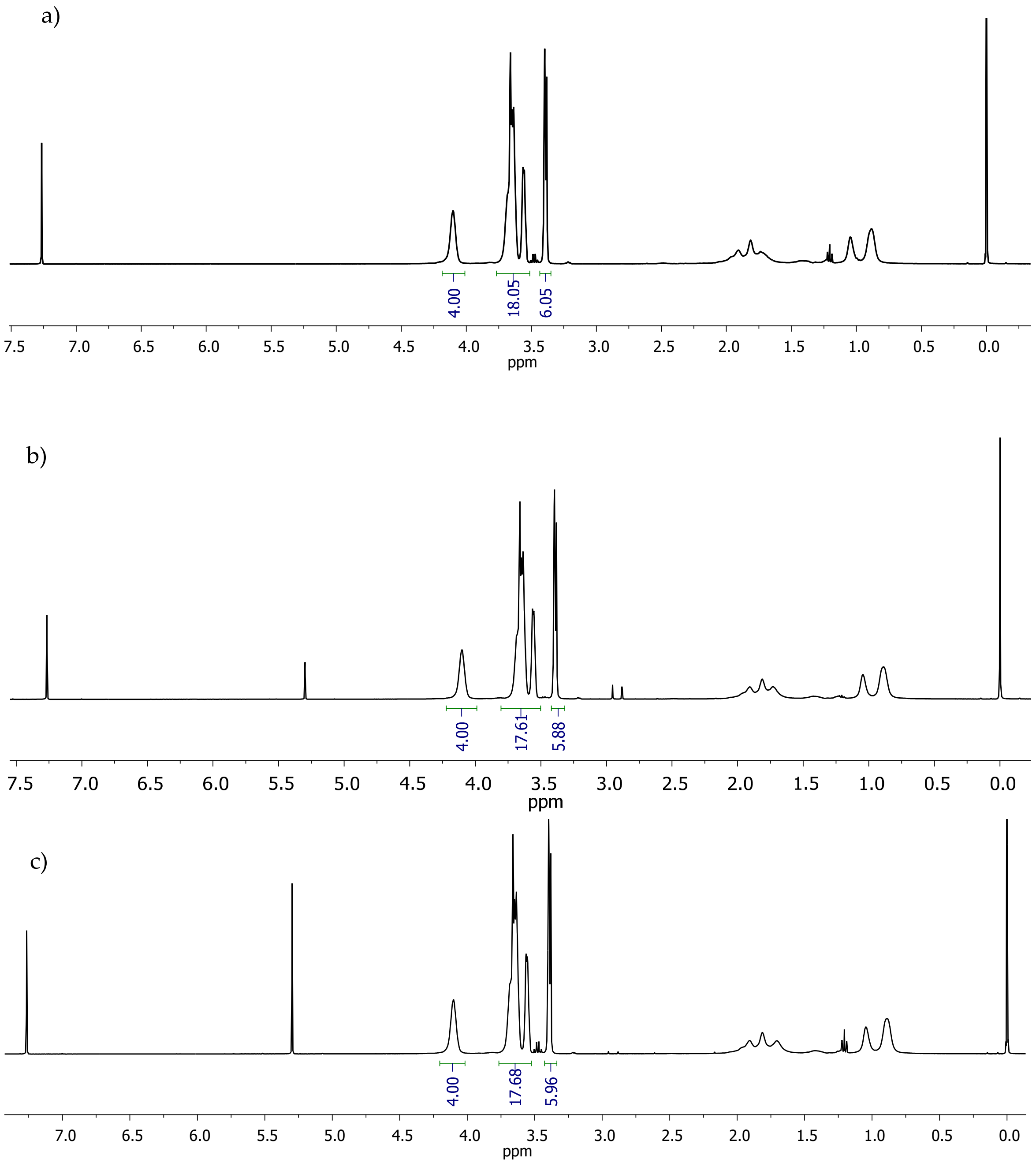

Figure S2. ^{1}H-NMR spectra (400 MHz) in $CDCl_3$ of P(DEGMA-*co*-OEGMA) copolymers: a) P1, b) P3, c) P4

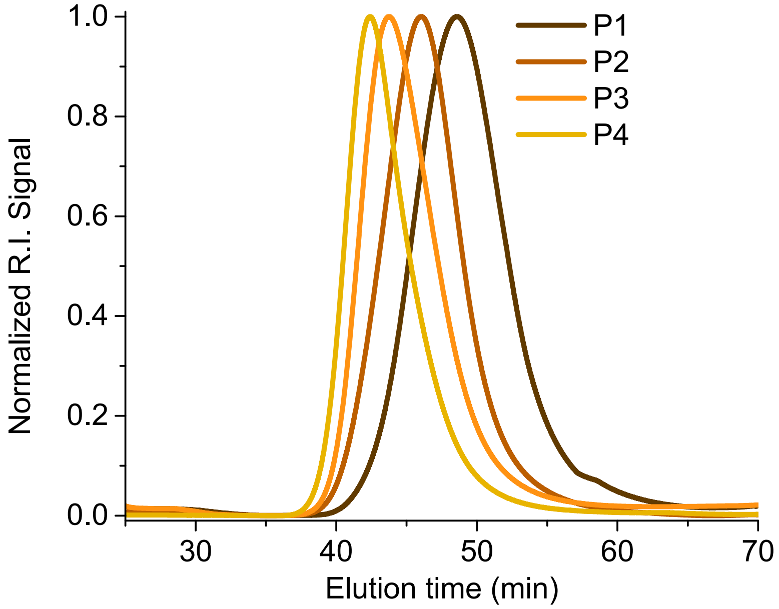

Figure S3. Normalized SEC traces (RI detector) of P(DEGMA-co-OEGMA) macro-CTA´s.

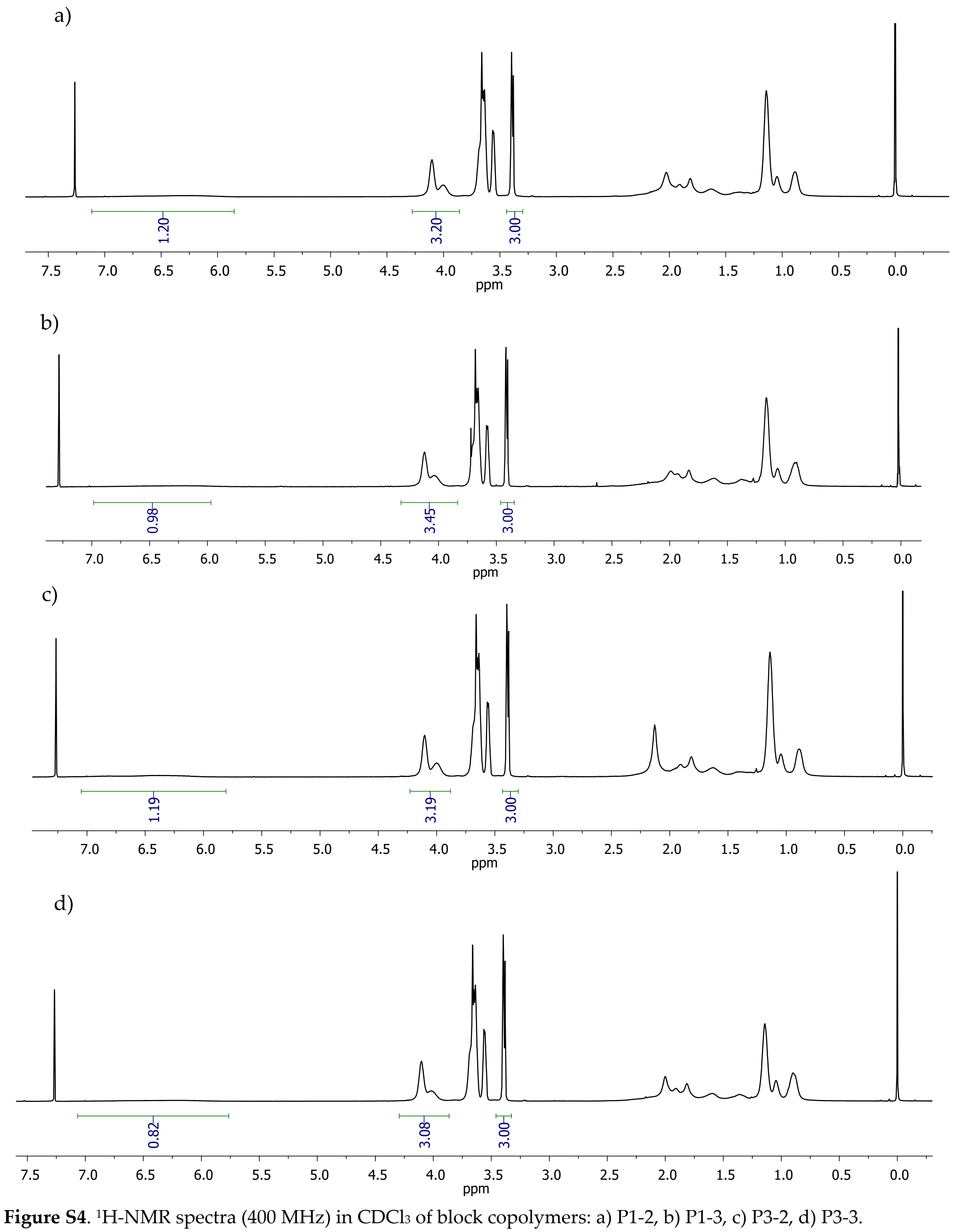

Figure S4. ^{1}H-NMR spectra (400 MHz) in $CDCl_3$ of block copolymers: a) P1-2, b) P1-3, c) P3-2, d) P3-3.

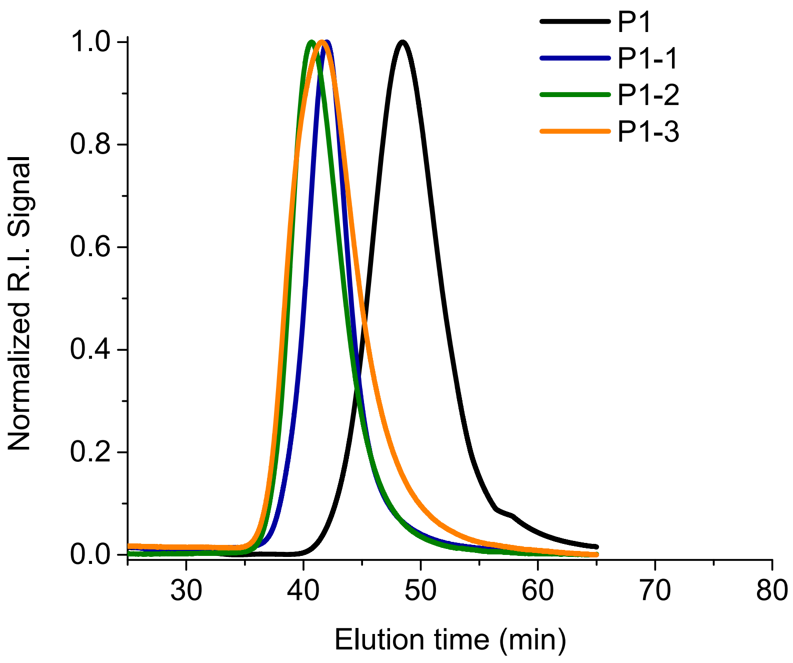

Figure S5. Normalized SEC traces (RI detector) in DMF of P(DEGMA-*co*-OEGMA)-*b*-PNIPAAm and P(DEGMA-*co*-OEGMA)-*b*-P(NIPAAm-*co*-BA) block copolymers.

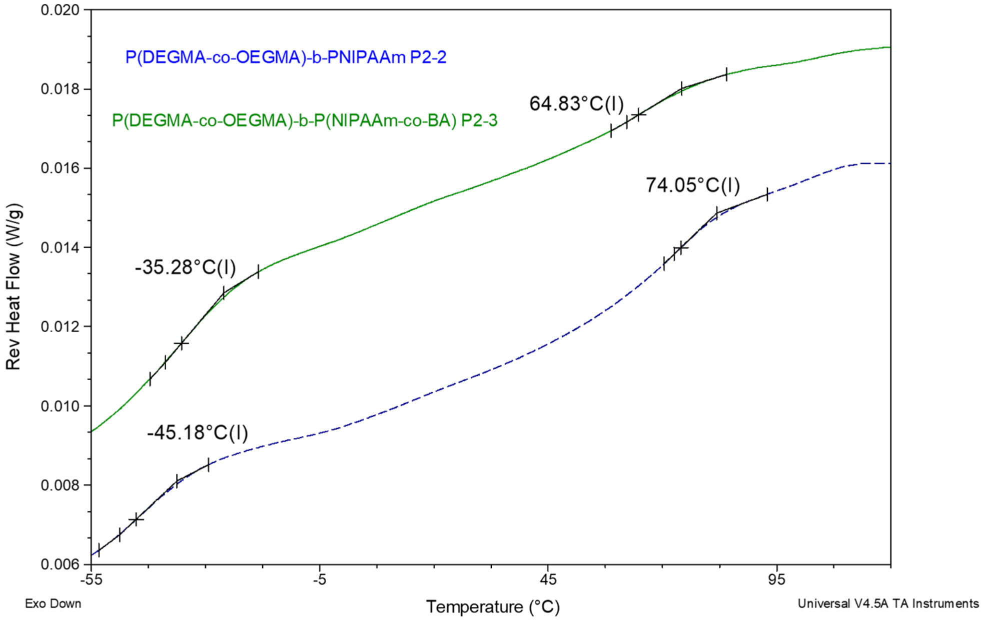

Figure S6. DSC thermograms of P(DEGMA-*co*-OEGMA)-*b*-PNIPAAm (P2-2) and P(DEGMA-*co*-OEGMA)-*b*-P(NIPAAm-*co*-BA) (P2-3) block copolymers.

a)

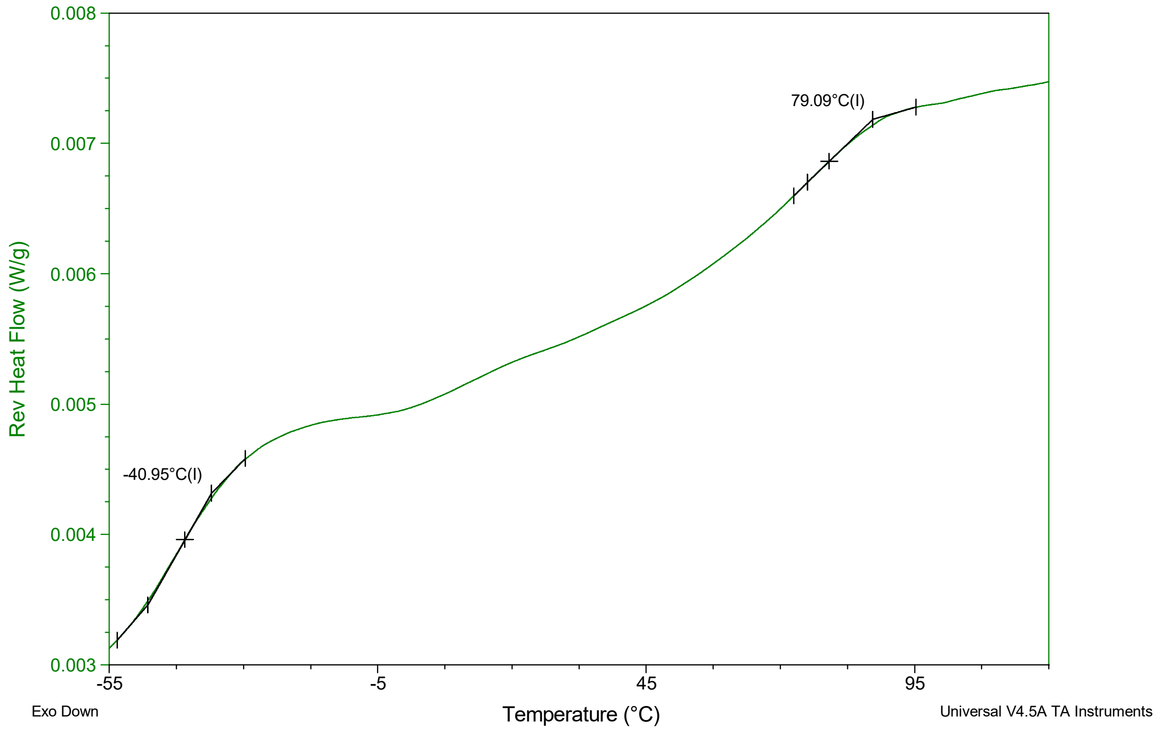

b)

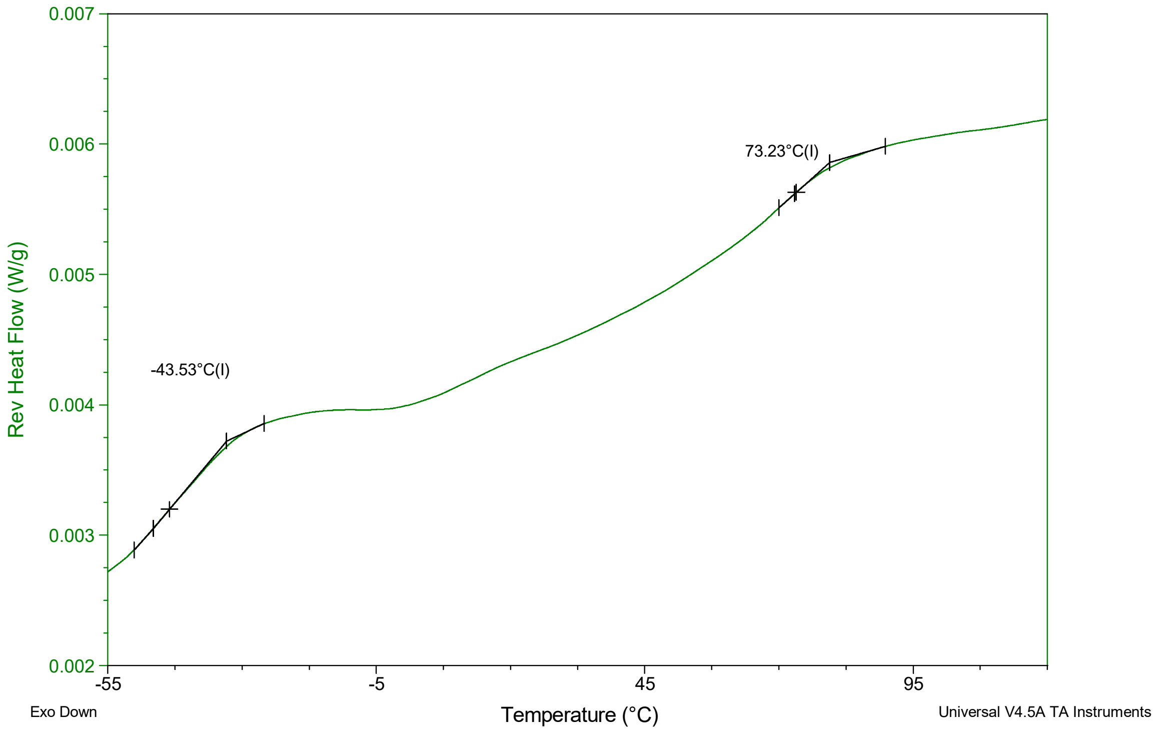

Figure S7. DSC thermograms of block copolymers: a) P1-2, b) P3-2.

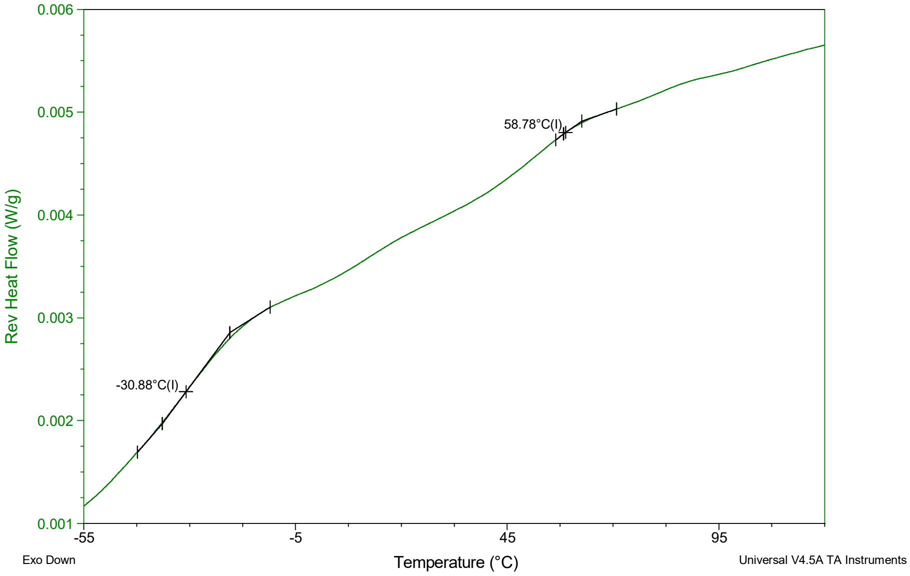

Figure S8. DSC thermograms of block copolymer P3-3.

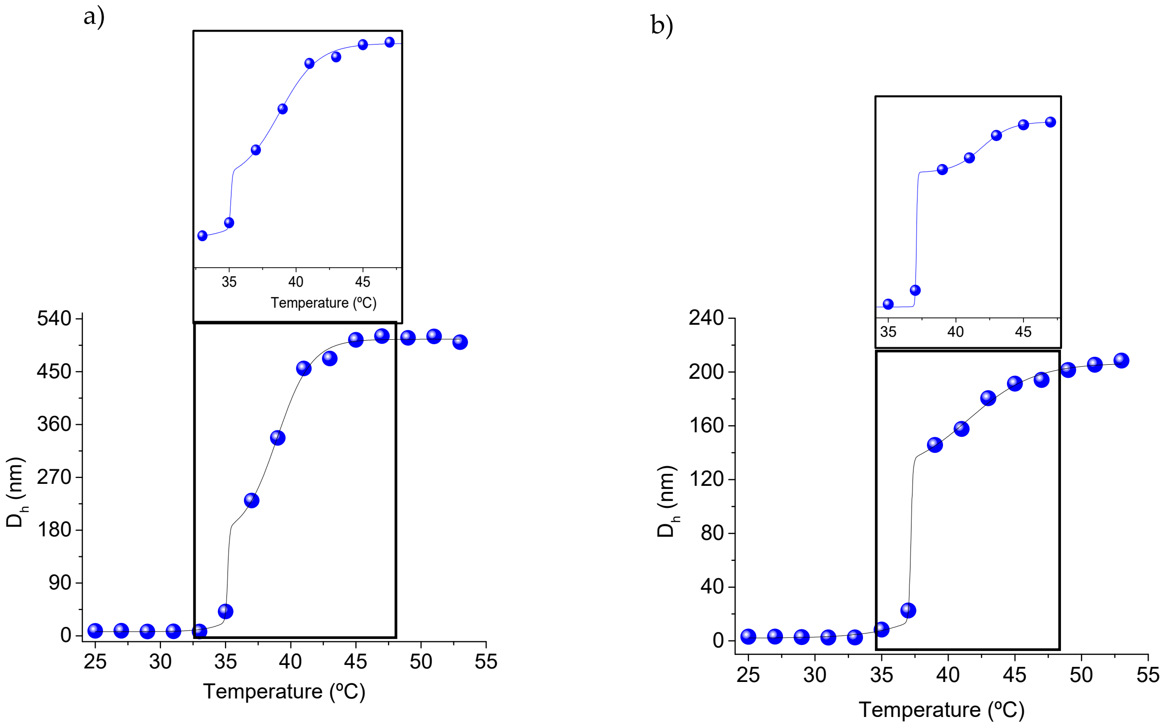

Figure S9. Determination of T_{cp} of block copolymers (1 mg mL^{-1}): a) P(DEGA-*co*-OEGMA)$_{47\%}$-*b*-PNIPAAm$_{53\%}$, b) P(DEGA-*co*-OEGMA)$_{45\%}$-*b*-PNIPAAm$_{55\%}$.

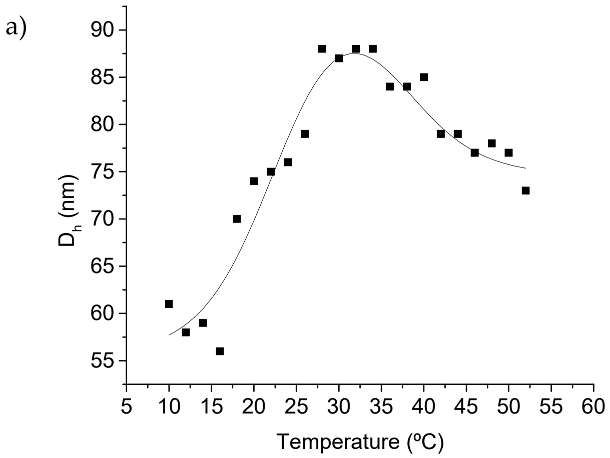

Figure S10. Determination of T_{cp} of block copolymers (1 mg mL^{-1}): a) P(DEGA-*co*-OEGMA)$_{50\%}$-*b*-P(NIPAAm$_{44\%}$-*co*-BA$_{6\%}$).

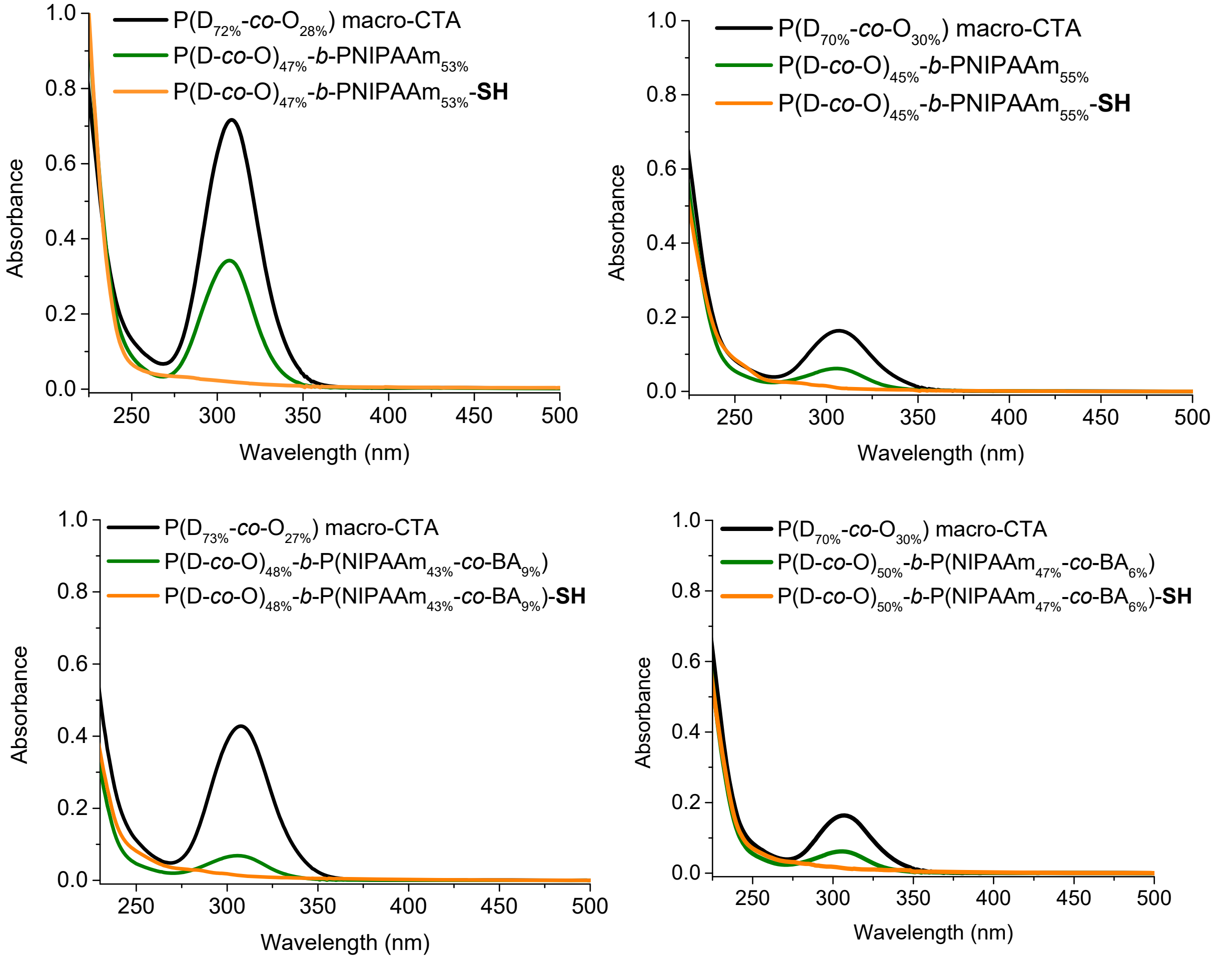

Figure S11. UV-Vis spectra (c=1 mg mL^{-1} in ethanol) of block copolymers and thiol-terminated block copolymers.

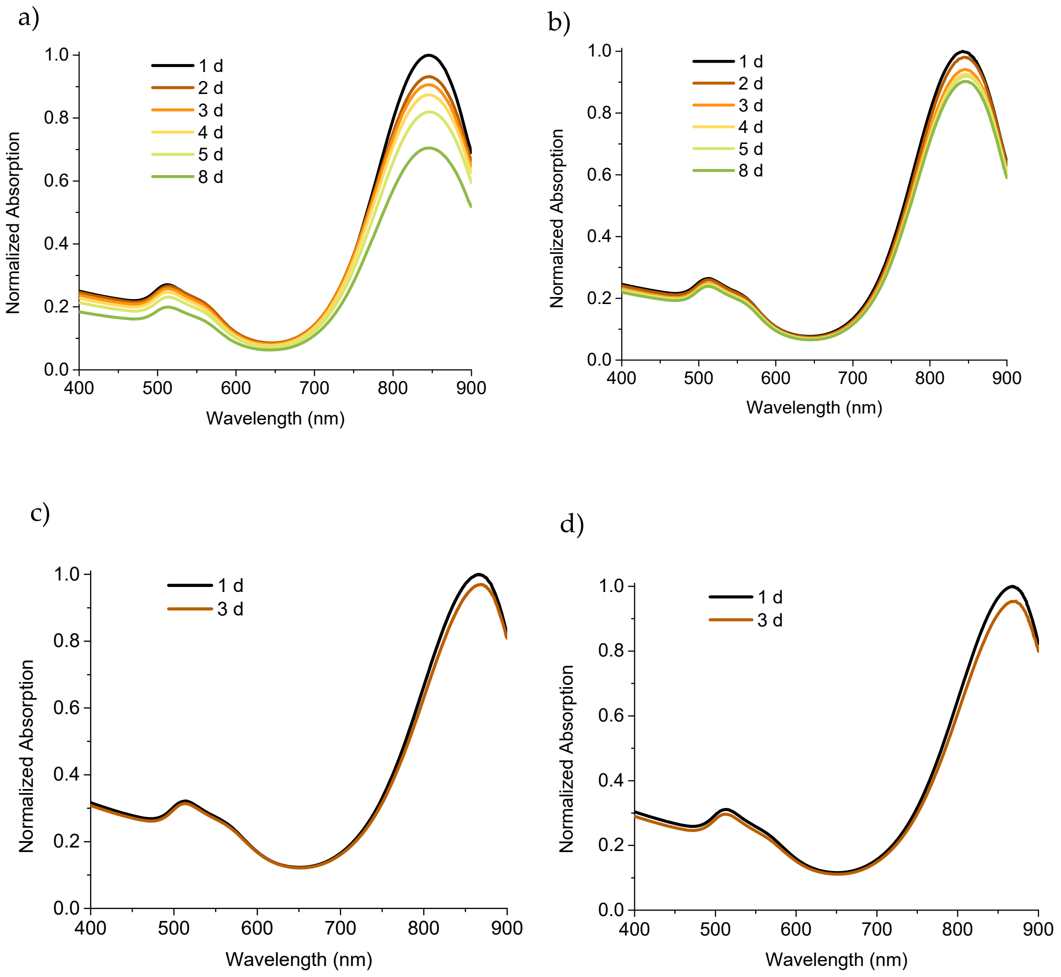

Figure S12. Optical stability against time: a) P(DEGMA-*co*-OEGMA)$_{47\%}$-*b*-PNIPAAm$_{53\%}$@GNRD, b) P(DEGMA-*co*-OEGMA)$_{45\%}$-*b*-PNIPAAm$_{55\%}$@GNRDs, c) P(DEGMA-*co*-OEGMA)$_{48\%}$-*b*-P(NIPAAm$_{43\%}$-*co*-BA$_{9\%}$)@GNRDs, d) P(DEGMA-*co*-OEGMA)$_{50\%}$-*b*-P(NIPAAm$_{44\%}$-*co*-BA$_{6\%}$)@GNRDs.

a)

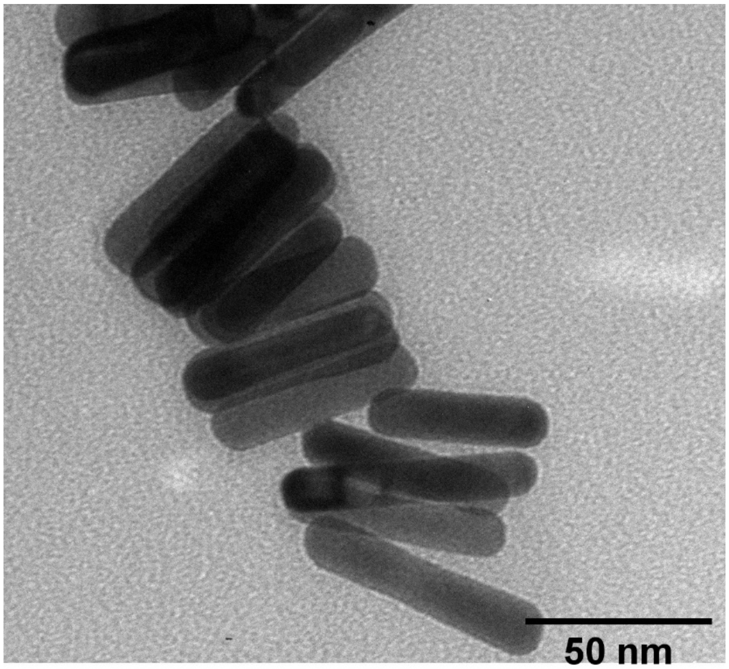

b)

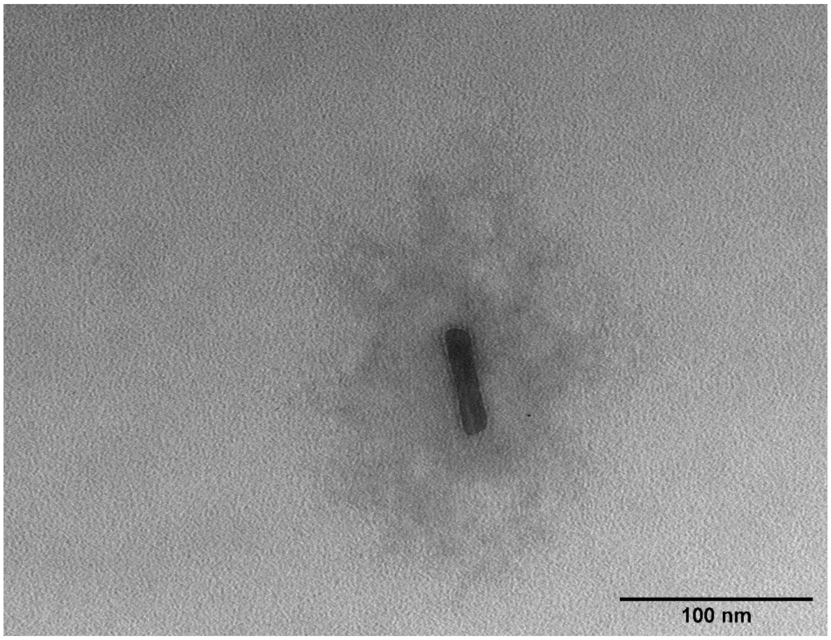

Figure S13. TEM micrographs of nanohybrids: a) P(DEGMA-*co*-OEGMA)$_{45\%}$-*b*-PNIPAAm$_{55\%}$@GNRDs, b) P(DEGMA-*co*-OEGMA)$_{50\%}$-*b*-P(NIPAAm$_{44\%}$-*co*-BA$_{6\%}$)@GNRDs.

Table S1. Aggregation of sample P(D-*co*-O)$_{37\%}$-*b*-PNIPAAm$_{63\%}$@GNRDs at different temperatures.

Sample	T (°C)	D_h (nm)	PDI	St Dev (d.nm)
P(D-*co*-O)$_{37\%}$-*b*-PNIPAAm$_{63\%}$@GNRDs	25 °C	85 & 3	0.821	46.11 & 0.9881
	37 °C	165	0.203	60.58
	40 °C	198	0.146	75.56

Table S2. Aggregation data of block copolymers@GNRDs at 37 °C.

Sample	D_h (nm)	PDI	St Dev (d.nm)
P(D-*co*-O)$_{47\%}$-*b*-PNIPAAm$_{53\%}$@GNRDs	247	0.184	76.10
P(D-*co*-O)$_{37\%}$-*b*-PNIPAAm$_{63\%}$@GNRDs	165	0.203	60.58
P(D-*co*-O)$_{45\%}$-*b*-PNIPAAm$_{55\%}$@GNRDs	135 & 16.1	0.282	55.79
P(D-*co*-O)$_{48\%}$-*b*-P(NIPAAm$_{43\%}$-*co*-BA$_{9\%}$)@GNRDs	788	1.00	109.3
P(D-*co*-O)$_{50\%}$-*b*-P(NIPAAm$_{44\%}$-*co*-BA$_{6\%}$)@GNRDs	190	0.119	60.89

a)

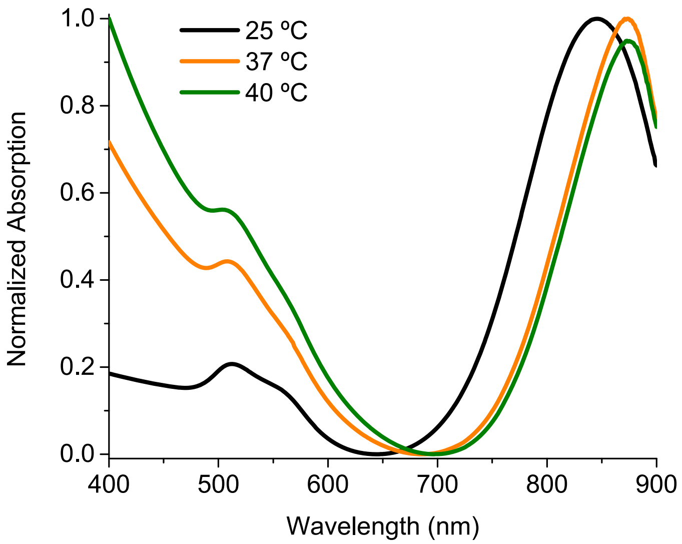

b)

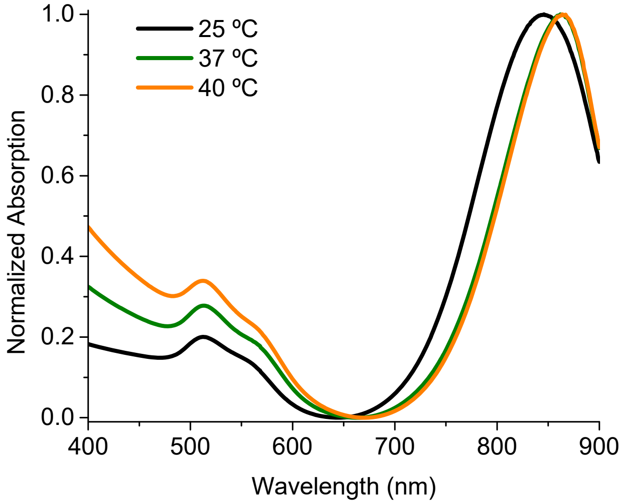

Figure S14. UV-Vis absorption spectra at 25 °C, 37 °C and 40 °C for nanohybrids: a) P(DEGMA-*co*-OEGMA)$_{47\%}$-*b*-PNIPAAm$_{53\%}$@GNRDs, b) P(DEGMA-*co*-OEGMA)$_{45\%}$-*b*-PNIPAAm$_{55\%}$@GNRDs.

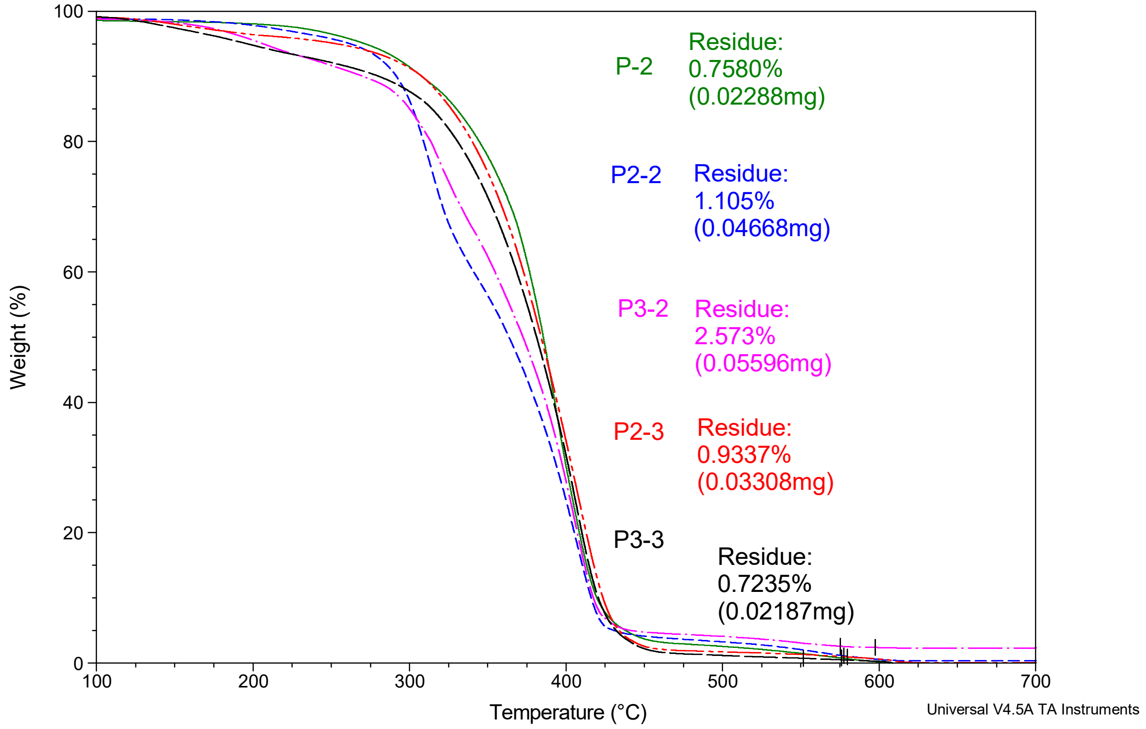

Figure S15. TGA-thermograms of block copolymers.
